# Supplementary figures and images for: A broadly neutralizing monoclonal antibody overcomes the mutational landscape of emerging SARS-CoV-2 variants of concern
Source: PLoS Pathog. 2022 Dec 12;18(12):e1010994. doi: 10.1371/journal.ppat.1010994 (PMC9779650; doi:10.1371/journal.ppat.1010994)

**A**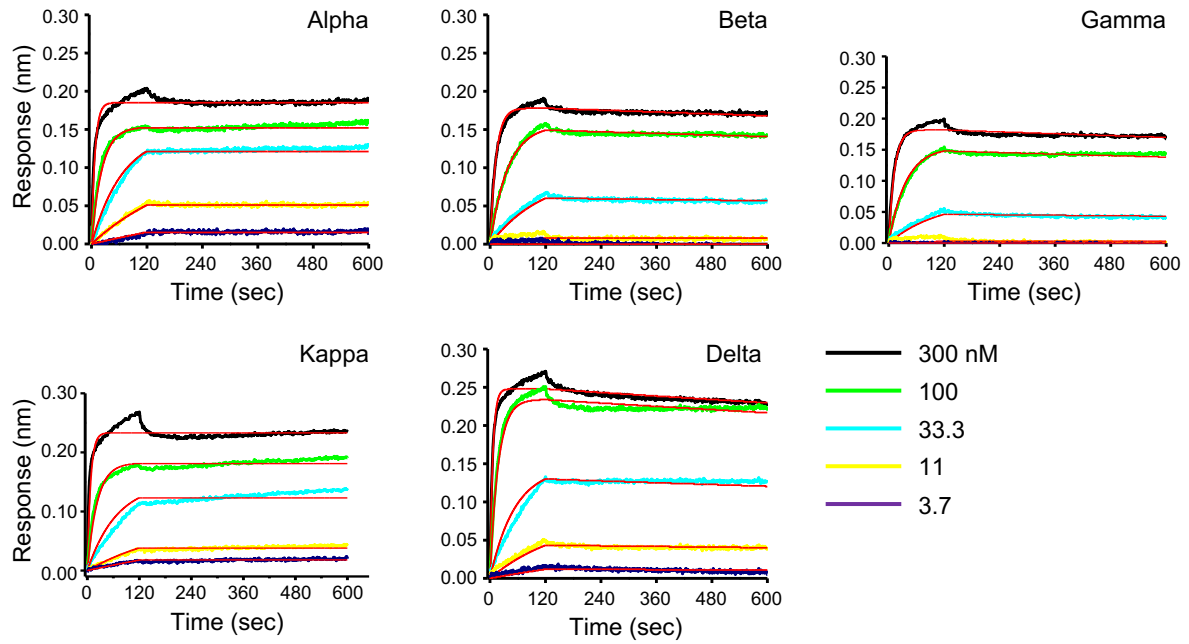**B**

| RBD construct | EC <sub>50</sub> |
|---------------|------------------|
| WA1/2020      | 0.0093 ± 0.0026  |
| Alpha         | 0.0099 ± 0.0018  |
| Beta          | 0.6002 ± 0.0247  |
| Gamma         | 0.1158 ± 0.0021  |
| Kappa         | 0.0131 ± 0.0142  |
| Delta         | 0.0121 ± 0.0014  |
| Omicron BA.1  | 0.2615 ± 0.0407  |

**C**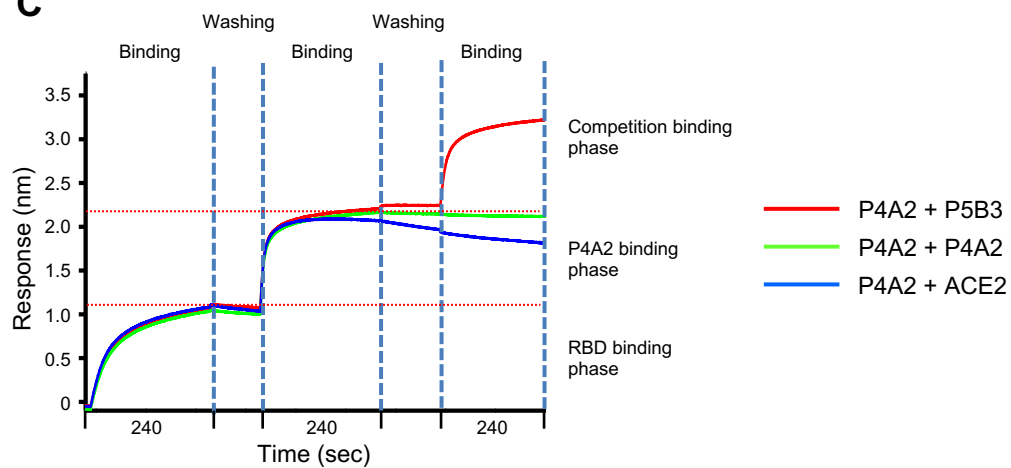

Supplement: S1 Fig — (A) P4A2 was immobilized on anti-mouse Fc biosensor and was tested using three-fold serial dilutions of RBD (starting with 300 nM and going down to 3.3 nM; the five concentrations tested are indicated). Data shown is after the reference was subtracted and aligned using Octet Data Analysis software v11.1 (Forte Bio). Curve fitting was done with a 1:1 binding model, and kon, koff and Kd values were calculated using a global fit. (B) Cross reactive binding potential (with half-maximal effective concentration, EC50) of P4A2 mAb to RBD proteins of different VOCs was tested by indirect ELISA. (C) The epitope specificity of P4A2 was evaluated for epitope competition using BLI. RBD-Fc was captured using anti-human Fc biosensor and saturated with P4A2 and the indicated mAbs at a concentration of 40 μg/ml and unbound P4A2 was washed, followed by incubation with 20 μg/ml of ACE2, P4A2 and P5B3 (binds to a topologically distinct, non-competing epitope and served as a control). No binding signal was observed for P4A2 and ACE2. (PDF) [file ppat.1010994.s001.pdf]

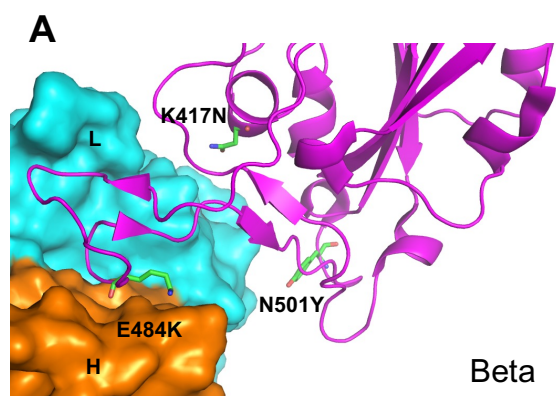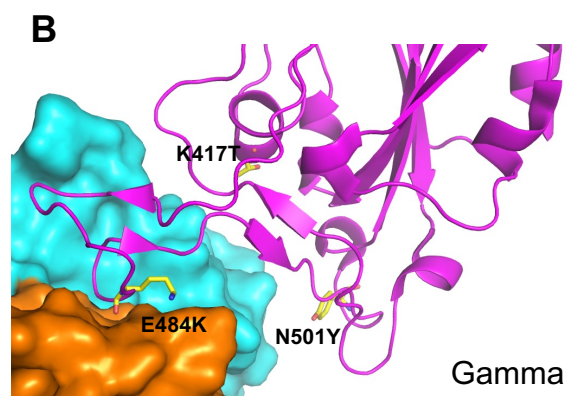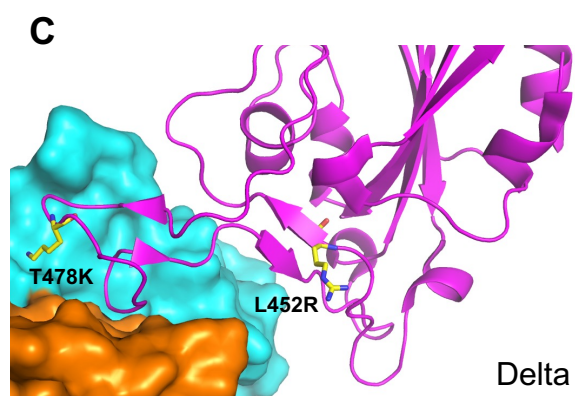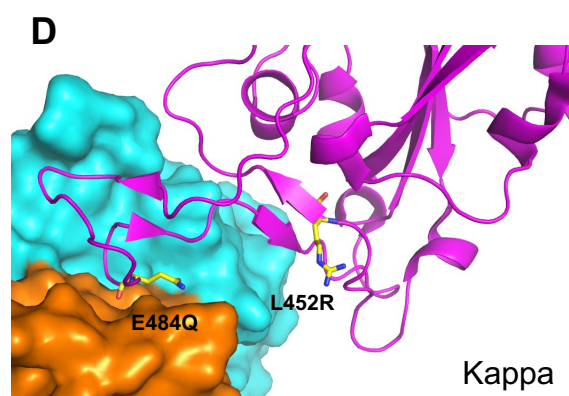

Supplement: S2 Fig — Using the crystal structure, computational models of P4A2 Fab in complex with the RBD from (A) Beta, (B) Gamma, (C) Delta, (D) Kappa and (E) BA.1 VOCs were generated. These models show that, for all the VOCs, there are no mutations in the residues that interact with the P4A2 through their side-chain and hence these mutations will not adversely impact P4A2 binding. E484 is mutated to Lys, Gln or Ala in some of the VOCs, but it forms interactions with the P4A2 Fab paratope through the backbone atoms and not through the side chain. (PDF) [file ppat.1010994.s002.pdf]

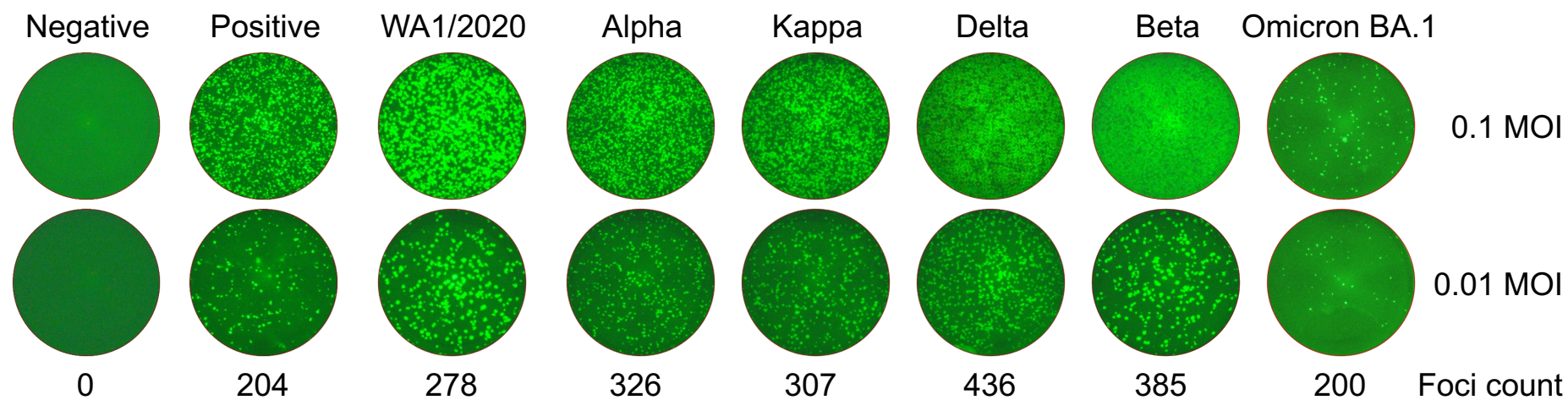

Supplement: S3 Fig — The binding of P4A2 to the spike proteins expressed on the surface of Vero E6 cell infected with various VOCs was assessed by immunofluorescence microscopy. Vero E6 cells were infected at a MOI of 0.1 and 0.01. The number of foci at MOI 0.1 were too numerous to be counted. The foci count for the MOI 0.01 are indicated. P4A2 was used as the primary antibody (diluted 1 in 2000) followed by anti-mouse Alexa 488 as the secondary antibody. The foci were counted using AID EliSpot 8.0 software. Representative images of the experiment performed in triplicates are shown. (PDF) [file ppat.1010994.s003.pdf]

**A**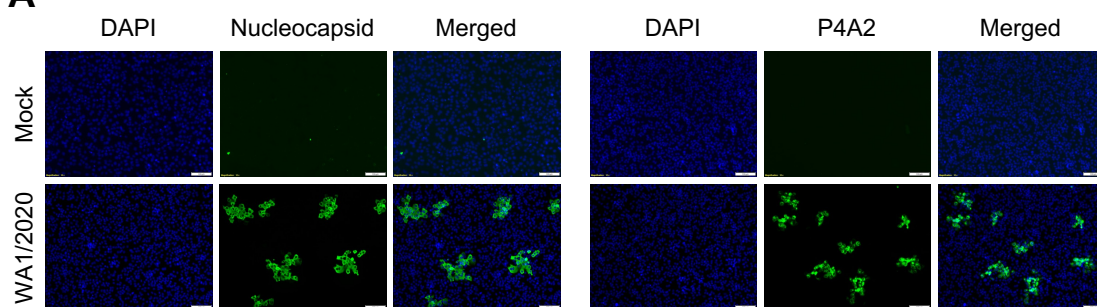**B**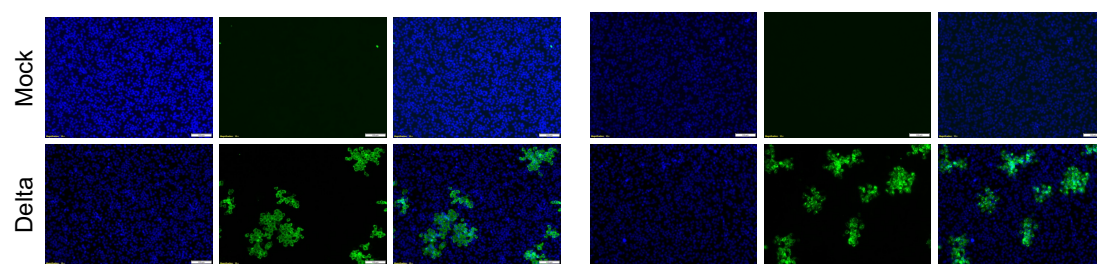**C**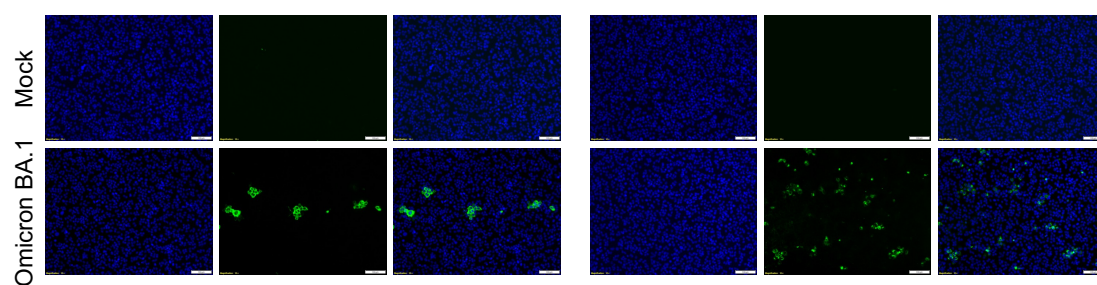**D**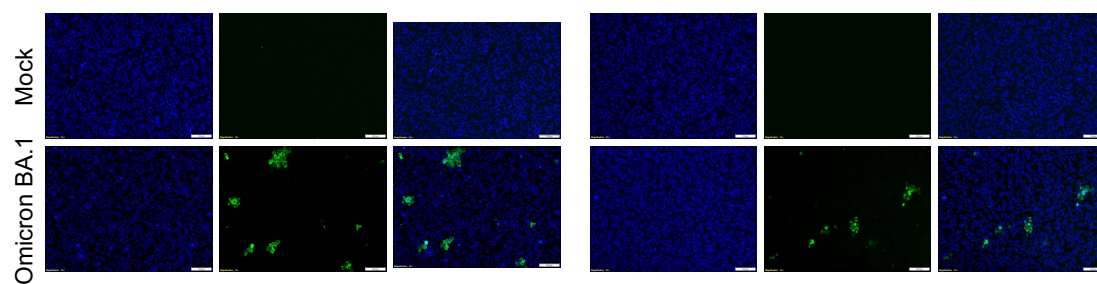

Supplement: S4 Fig — Vero E6 cells were infected with Wuhan (A), Delta (B) and BA.1 variant of SARS-CoV-2 at a MOI of 0.1 (C) and 0.01 (D). Cells were fixed with 7.4% formaldehyde 32 h following infection, stained with mAb P4A2 and observed under an immunofluorescence microscope. DAPI was used to stain nuclei. Images were captured at a magnification of 10×. Scale bar represents 100 μm. (PDF) [file ppat.1010994.s004.pdf]

**A**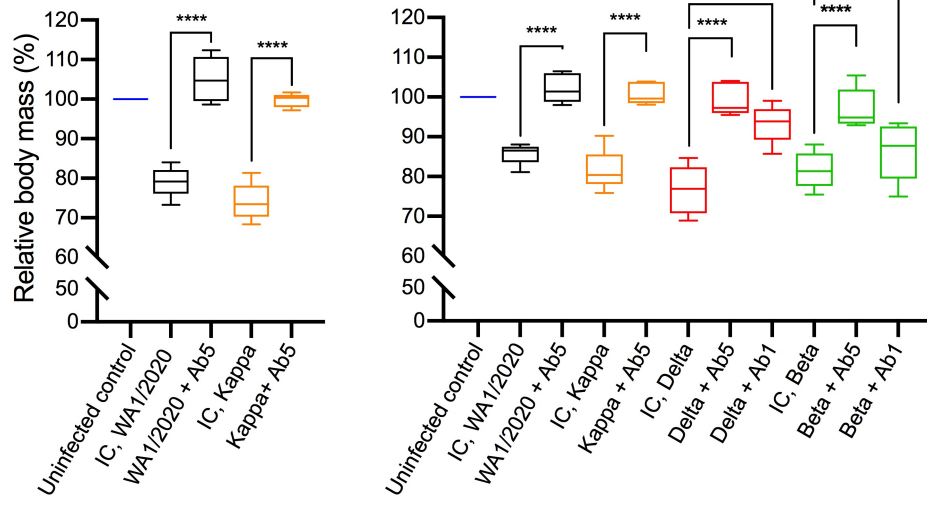**B**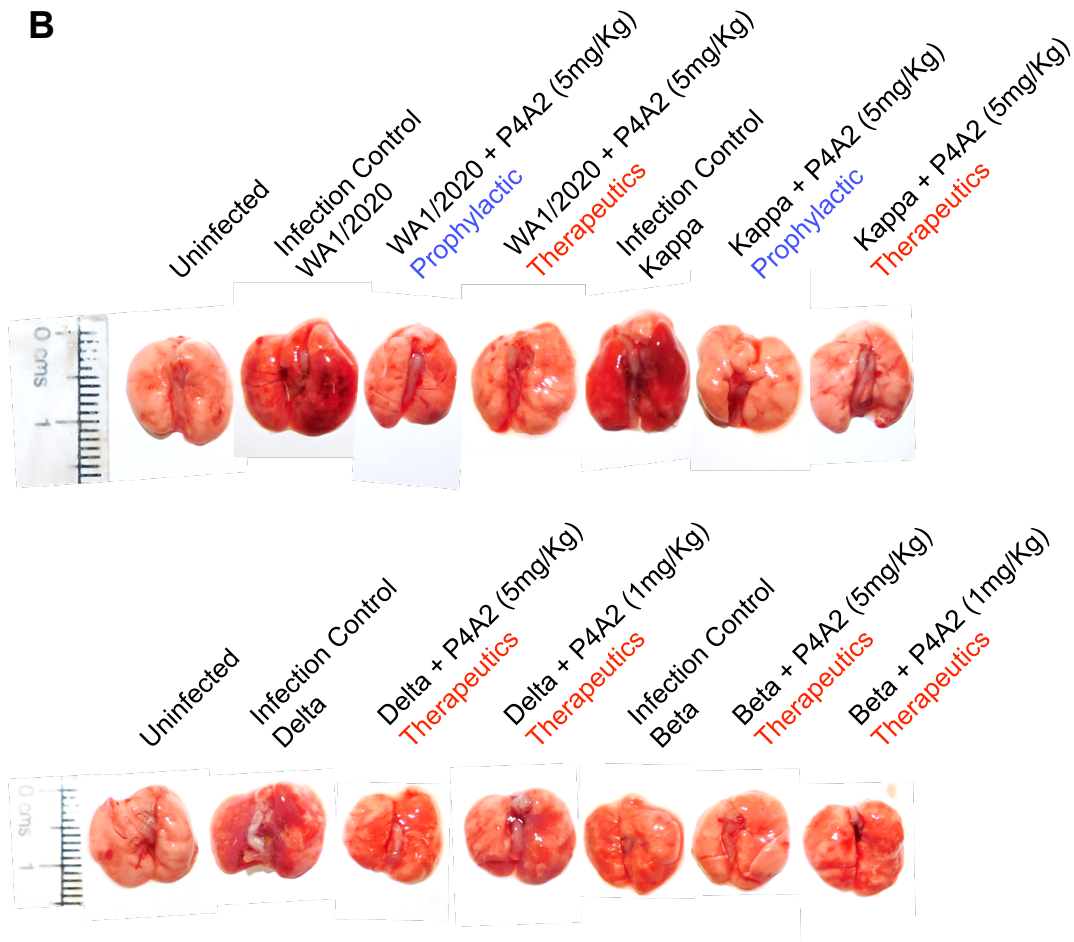

Supplement: S5 Fig — (A) Body mass of mice from each group (both prophylactic and therapeutic) was recorded for 6 days post-infection. The Y-axis represents the body mass of the mouse relative to the body mass of the same mouse recorded on day 0 (normalised to 100) (dpi, days post-infection). (B) Animal challenge experiment performed with prophylactic or therapeutic intervention of P4A2 antibody. Briefly, animals challenged with Wuhan, Kappa, Delta or Beta SARS-CoV-2 strain (105 pfu/ mice) were given prophylactic (1 day prior to challenge) or therapeutic dose (12 h post challenge) and representative images of the excised lung showing inflammation and pneumonitis. (PDF) [file ppat.1010994.s005.pdf]

**A**

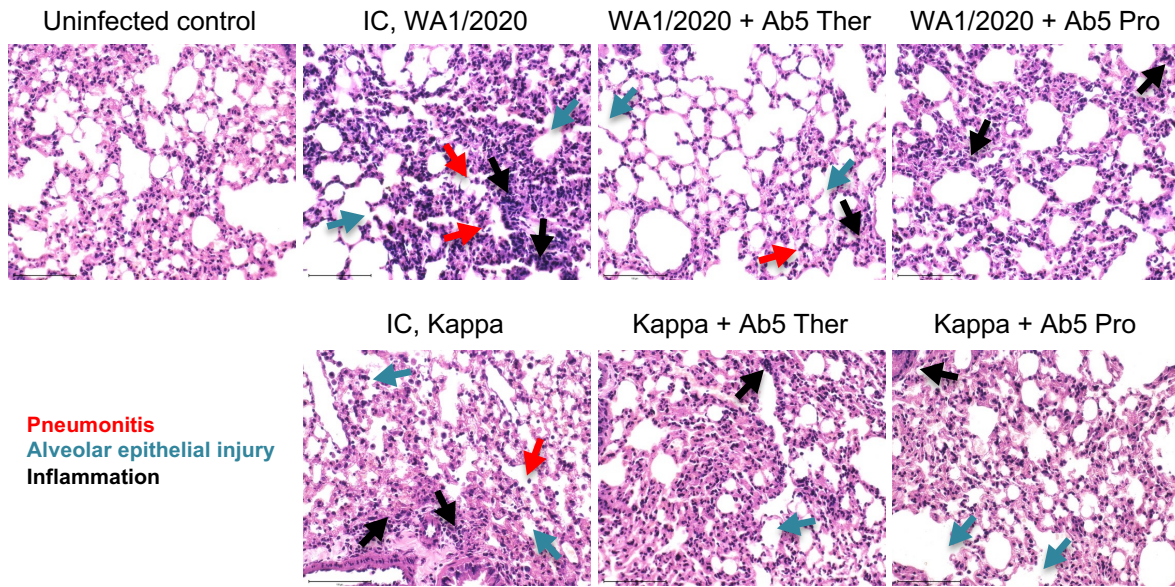

**B**

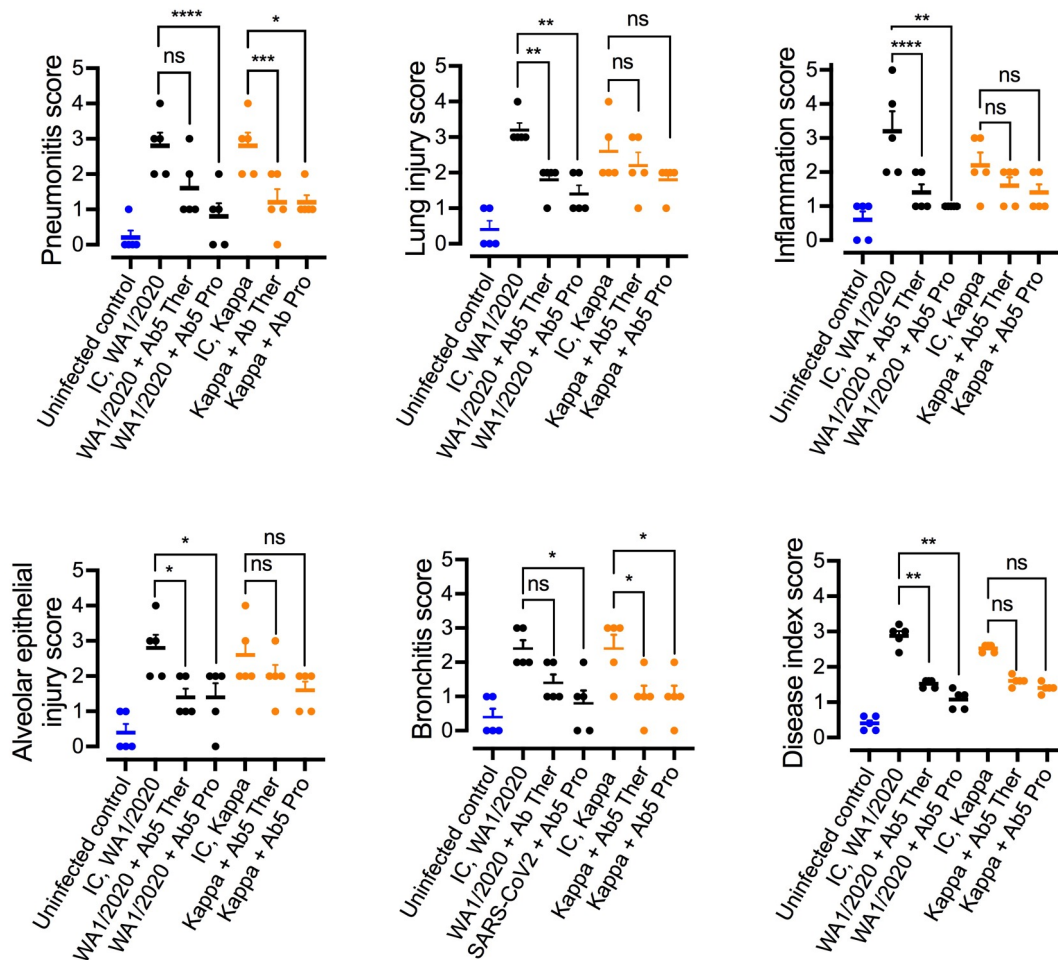

Supplement: S6 Fig — Animals challenged with SARS-CoV-2 with or without P4A2 antibody were euthanized on day 6 post-infection. The left lower lobe of their lung were fixed in 10% formalin solution and used for H & E staining. (A) Representative images of transverse section of the lung showing pneumonitis (magnification = 40×; red arrow), alveolar epithelial injury (blue arrow) and inflammation (black arrow). The stained sections were assessed by blinded-trained histologist on the scale of 0–5 (where 0 represents no feature, while 5 represents the maximum score). (B) The histological scores for each pulmonary pathology was plotted as mean ± SEM. The disease index score was calculated by taking the average score of all pulmonary pathologies (i. e. pneumonitis, alveolar epithelial injury and inflammation). Ther, therapeutic; Pro, prophylactic. NS, not statistically significant. (PDF) [file ppat.1010994.s006.pdf]

**A**

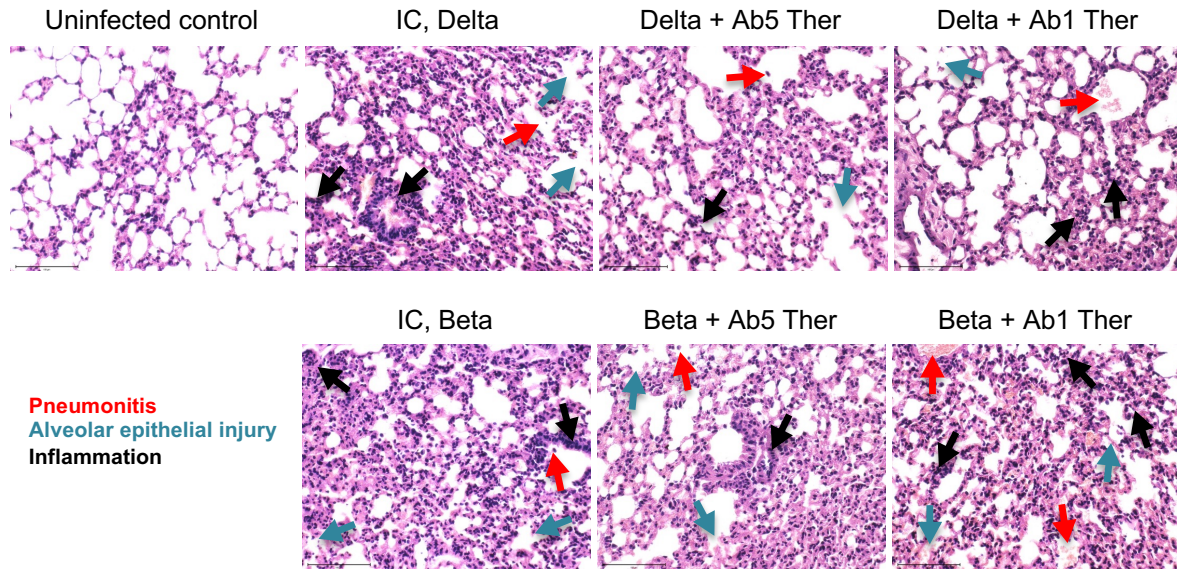

**B**

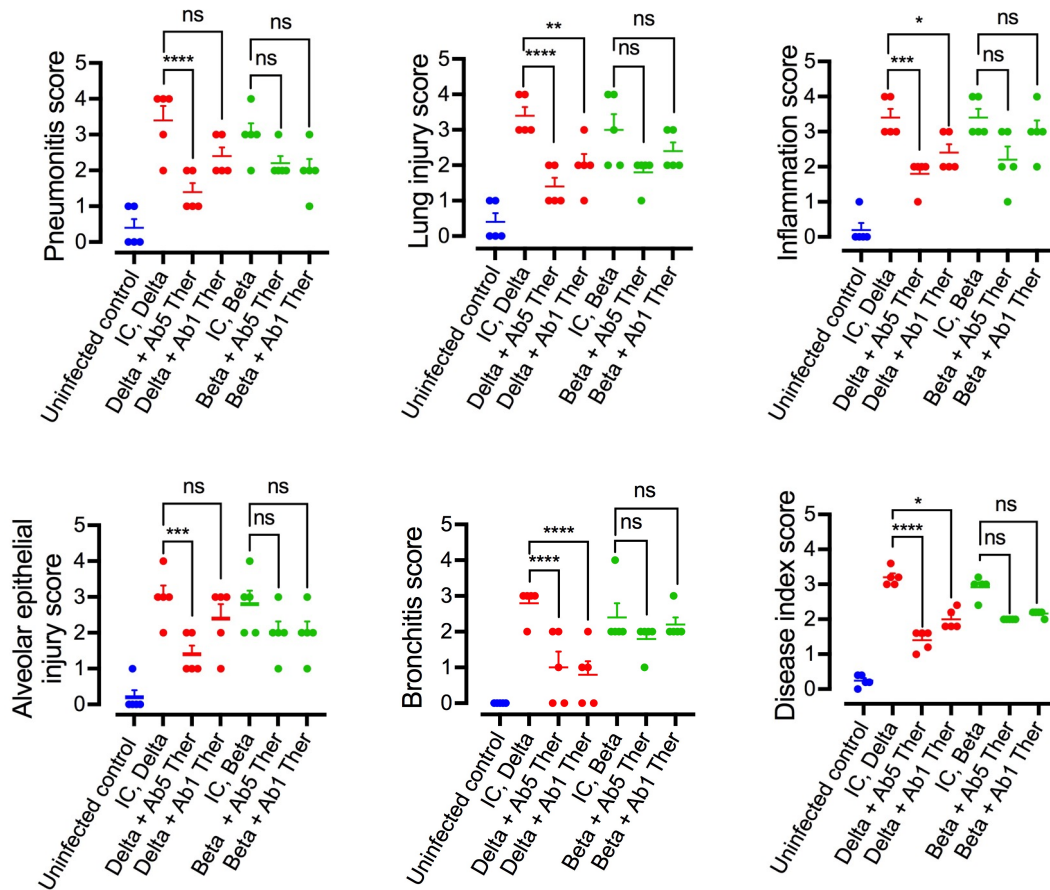

Supplement: S7 Fig — Lung samples from euthanized animals 6 days post challenge were fixed in 10% formalin solution and stained with H & E. (A) Representative transverse section of the H & E stained lung images at 40× magnification showing pneumonitis (red arrow), alveolar epithelial injury (blue arrow) and inflammation (black arrow). The stained sections were assessed by blinded-trained histologist on the scale of 0–5 (where 0 represents no feature, while 5 represents the maximum score). (B) The histological scores for each pulmonary pathology was plotted as mean ± SEM. The disease index score was calculated by taking the average score of all pulmonary pathologies. Ther, therapeutic; NS, not statistically significant. (PDF) [file ppat.1010994.s007.pdf]

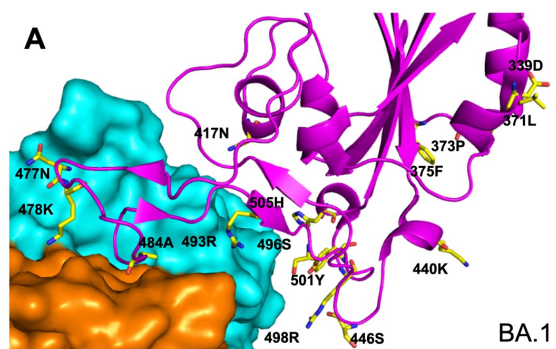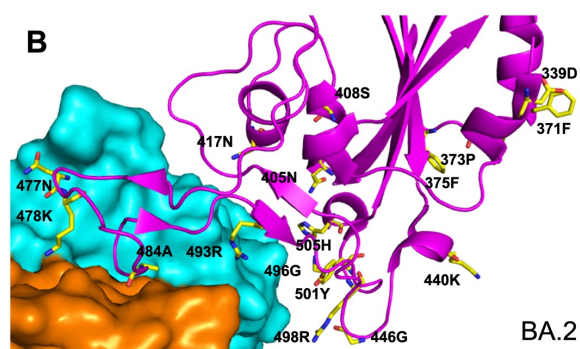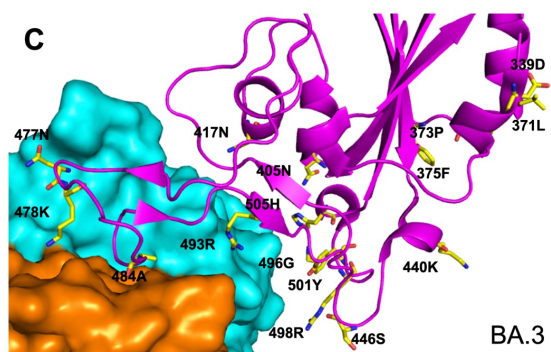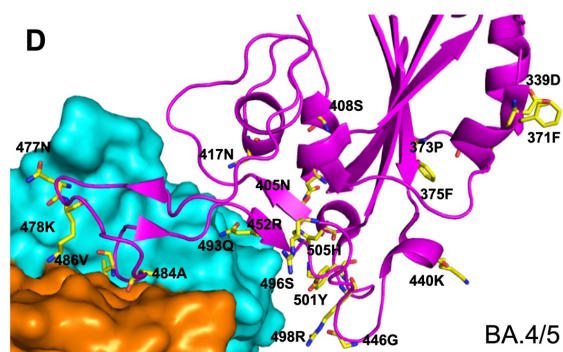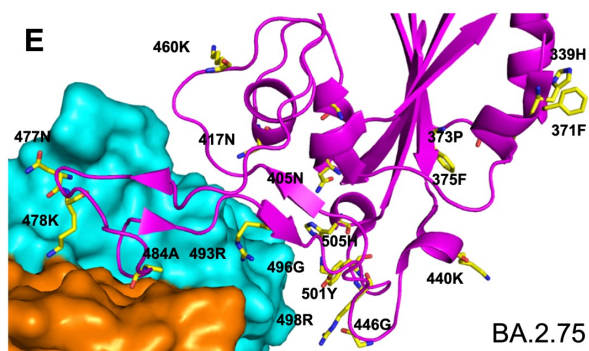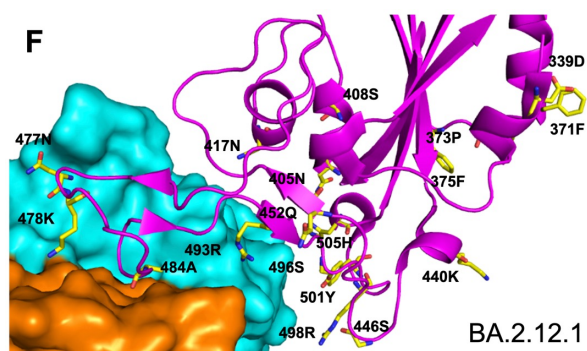

Supplement: S10 Fig — Computational models of P4A2 Fab in complex with spike-RBD from different Omicron lineages BA.1, BA.2, BA.3, BA.4/5, BA.2.75 and BA.2.12.1. (PDF) [file ppat.1010994.s010.pdf]

P4A2

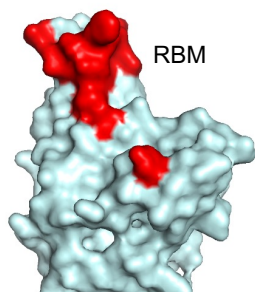

87G7

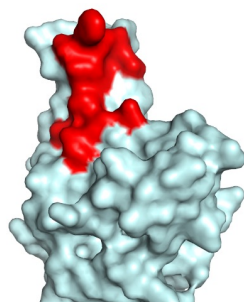

510A5

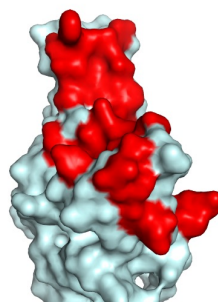

ABP-310

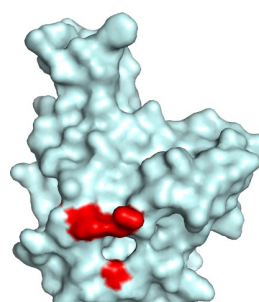

B8-dlaG2

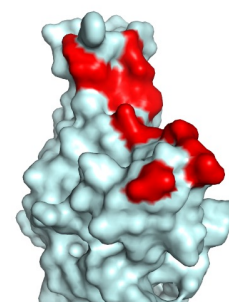

CAB-A17

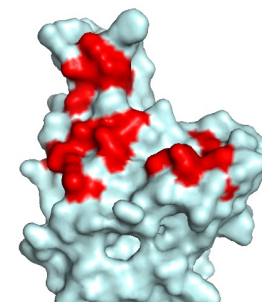

Cov2-2196

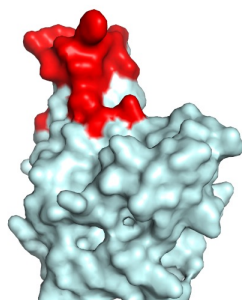

LY-CoV1404

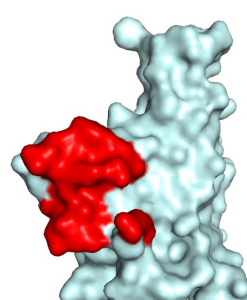

NCV2SG48

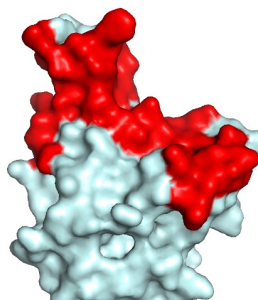

NCV2SG53

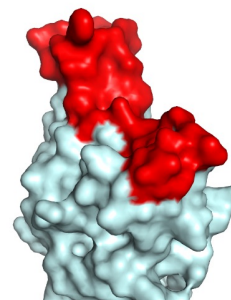

S2E12

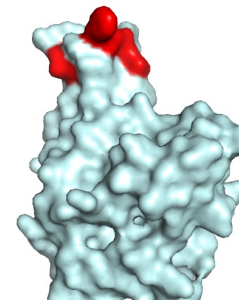

S2K146

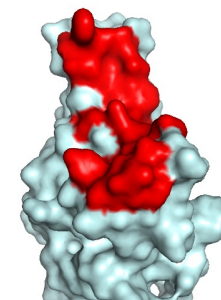

S2X324

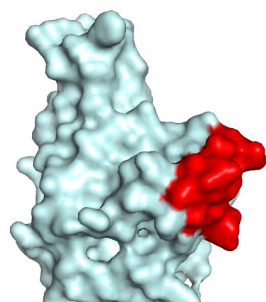

VacW-209

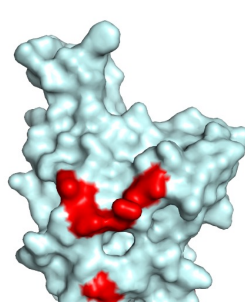

XGv051

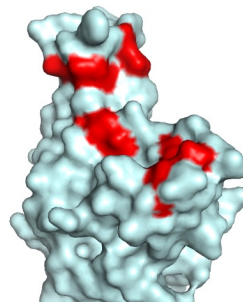

XGv264

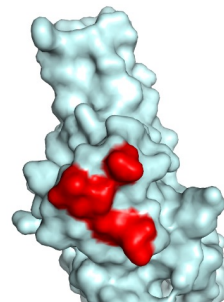

XGV286

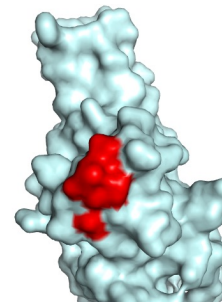

ZWD12

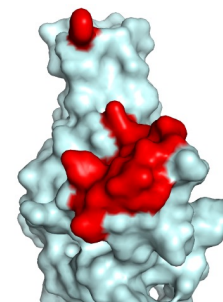

S309

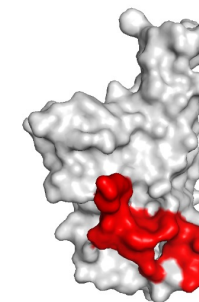

Supplement: S11 Fig — The surface of the spike-RBD is displayed and the epitope for different mAbs are shown in red colour. The mAbs JMB2002 and S3H3 are not shown here because the epitopes for these two antibodies are outside the RBD. The epitopes of 87G7, 510A5, Cov2-2196, NCV2SG48, NCV2SG53, S2E12, S2K146 and ZWD12 showed some overlap with that of P4A2 but only P4A2 mAb possesses a hydrophobic cleft into which the 486Phe residue is buried. Based on available information, P4A2 forms multiple interactions with its cognate epitope on spike-RBD and multiple residues present in this epitope are critical for interaction with ACE2. (PDF) [file ppat.1010994.s011.pdf]
