# Supplementary material for: A broadly neutralizing monoclonal antibody overcomes the mutational landscape of emerging SARS-CoV-2 variants of concern
Source: PLoS Pathog. 2022 Dec 12;18(12):e1010994. doi: 10.1371/journal.ppat.1010994 (PMC9779650; doi:10.1371/journal.ppat.1010994)
Supplement: S8 Fig — (A) SARS-CoV-2 neutralizing antibodies were used as the positive control [two-fold dilution series starting at 1:20 and ending at 1:640 (highlighted in green)]. Pre-defined virus dilution (virus only control; wells 7 through 11 in rows A and B to get at least 60–200 FFU/well) and only medium (no virus control; added in well 12 in rows A and B) served as the control. P4A2 (rows C and D), Sotrovimab (rows E and F) and CR30322 (rows G and H) as two-fold serial dilutions were used in the experiment. The antibody neutralization assays against SARS-CoV-2 Delta variant (B.1.617.2) (B) and Omicron variant (B.1.1.529) (C) was done similarly. (PDF) [file ppat.1010994.s008.pdf]

A

<table>
<tr><th></th><th></th><th>1</th><th>2</th><th>3</th><th>4</th><th>5</th><th>6</th><th>7</th><th>8</th><th>9</th><th>10</th><th>11</th><th>12</th></tr>
<tr><td></td><td>A</td><td rowspan="2">1:20</td><td rowspan="2">1:40</td><td rowspan="2">1:80</td><td rowspan="2">1:160</td><td rowspan="2">1:320</td><td rowspan="2">1:640</td><td rowspan="2" colspan="5">Virus only control</td><td rowspan="2">No virus control</td></tr>
<tr><td></td><td>B</td></tr>
<tr><td rowspan="6">Monoclonal Ab (µg/mL)
P4A2 (C–D)
Sotrovimab (E–F)
CB30322 (G–H)</td><td>C</td><td rowspan="6">5</td><td rowspan="6">2.5</td><td rowspan="6">1.25</td><td rowspan="6">0.625</td><td rowspan="6">0.3125</td><td rowspan="6">0.1562</td><td rowspan="6">0.0781</td><td rowspan="6">0.0390</td><td rowspan="6">0.0195</td><td rowspan="6">0.0097</td><td rowspan="6">0.0048</td><td rowspan="6">0.0024</td></tr>
<tr><td>D</td></tr>
<tr><td>E</td></tr>
<tr><td>F</td></tr>
<tr><td>G</td></tr>
<tr><td>H</td></tr>
</table>

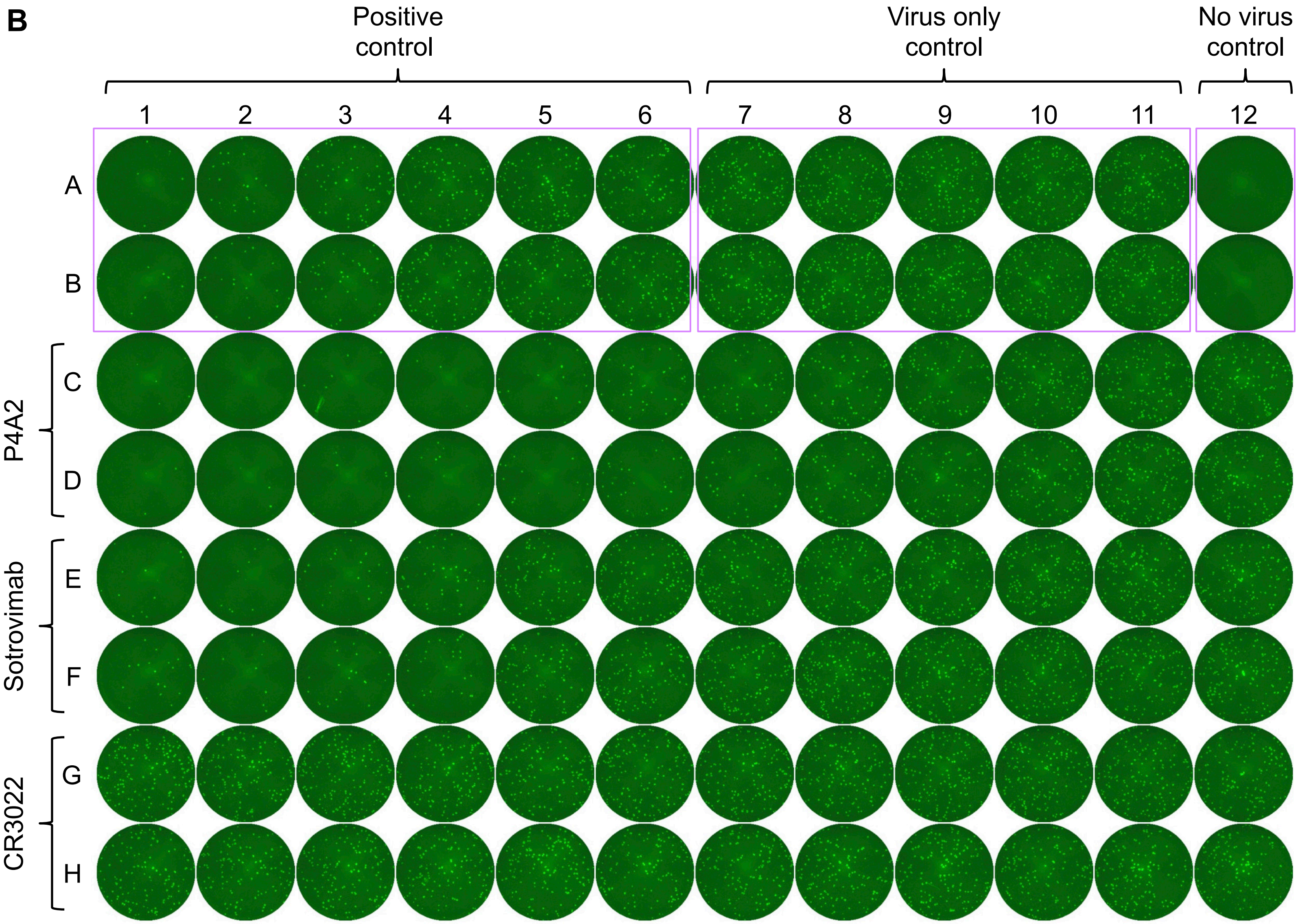
B
Positive control
Virus only control
No virus control
1
2
3
4
5
6
7
8
9
10
11
12
A
B
C
D
E
F
G
H
P4A2
Sotrovimab
CR3022

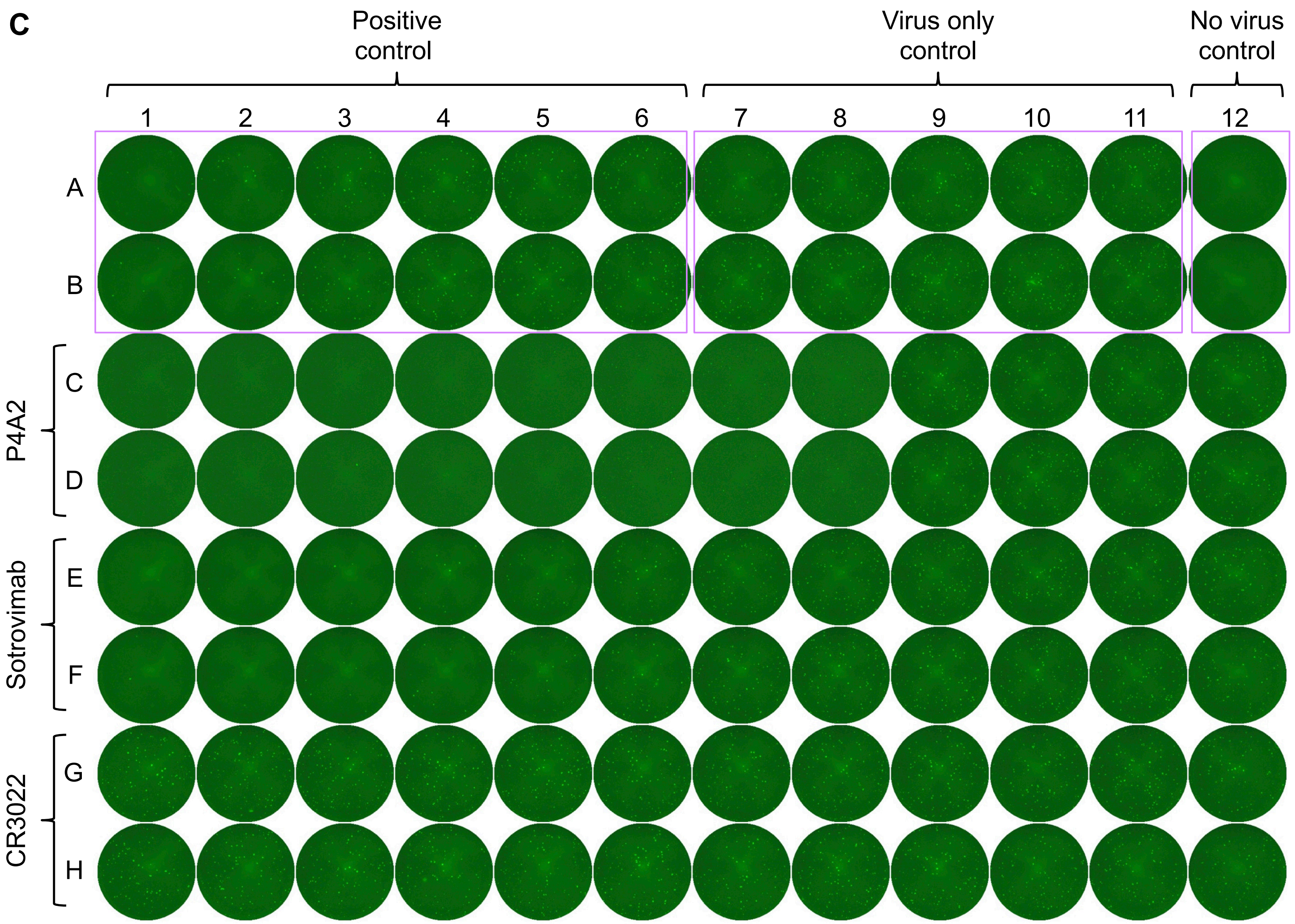
C
Positive control
Virus only control
No virus control
1
2
3
4
5
6
7
8
9
10
11
12
A
B
C
D
E
F
G
H
P4A2
Sotrovimab
CR3022
