# Supplementary material for: A broadly neutralizing monoclonal antibody overcomes the mutational landscape of emerging SARS-CoV-2 variants of concern
Source: PLoS Pathog. 2022 Dec 12;18(12):e1010994. doi: 10.1371/journal.ppat.1010994 (PMC9779650; doi:10.1371/journal.ppat.1010994)

A

P4A2-H

Q V Q L Q Q S G A E L V K P G A S V K L S C K A S G F T F T R Y S I Y W M
K Q R P G Q G L E W I G E I N P S T G D T N F N E K F K S K A T L T V D K
S S T T A Y M Q L S S L T S E D S A V Y Y C T R S T G Y W G Q G T L V T V
S A

P4A2-L

D I V L T Q S P A S L A V S L G Q R A T I S C R A S E S V E Y H G T I L M
Q W F Q Q K P G Q P P R L L I Y A A S N V D S G V P A R F S G S G S G T D
L S L N I H P V E E D D I A M Y F C Q Q S R K V P Y T F G G G T K L E I K

B

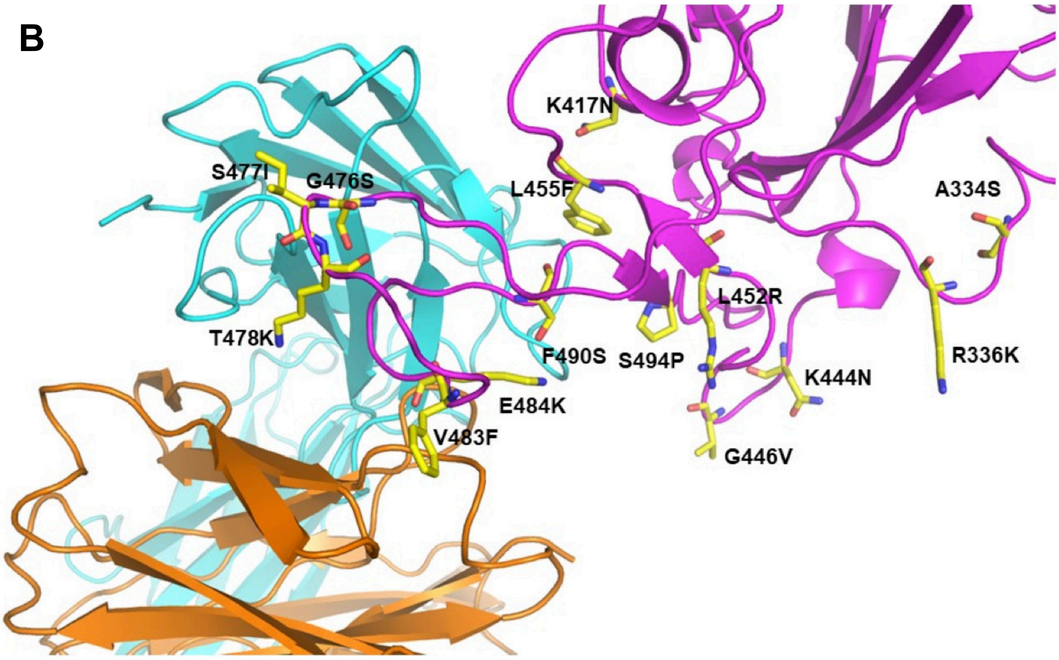

Supplement: S9 Fig — (A) Amino acid sequence of the variable regions of heavy and light chain of mAb P4A2 are denoted as P4A2-H and P4A2-L, respectively. Framework regions are shown in black, whereas the complementary determining region 1, 2 and 3 are highlighted in red, blue and green, respectively. (B) The mutations predicted by Maher et al. (2022) [5] are displayed in stick representation and coloured according to element. The spike-RBD, heavy and light chains of P4A2 Fab are shown in magenta, orange and cyan, respectively. None of the predicted mutations of the spike-RBD overlap with the residues that interact with P4A2 Fab and therefore it is possible that these mutations may not reduce the ability of P4A2 to neutralize the corresponding new variants of SARS-CoV-2. (PDF) [file ppat.1010994.s009.pdf]
