# Supplementary material for: A broadly neutralizing monoclonal antibody overcomes the mutational landscape of emerging SARS-CoV-2 variants of concern
Source: PLoS Pathog. 2022 Dec 12;18(12):e1010994. doi: 10.1371/journal.ppat.1010994 (PMC9779650; doi:10.1371/journal.ppat.1010994)
Supplement: S1 Table — (DOCX) [file ppat.1010994.s012.docx]

**Table S1: Comparative neutralization potential of P4A2 mAb with the clinical approved or under development therapies**

| mAb | Developer | Ancestral | Delta | Omicron BA.1 | Reference |
| --- | --- | --- | --- | --- | --- |
| Sotrovimab | Vir Biotechnology/ GSK | 372 | 369 | 1,059 | [1,2] |
| Casirivimab | Regeneron | 27 | 2.2 | >9,000 | [1,2] |
| Imdevimab | Regeneron | 25 | 5.7 | >9,000 | [1,2] |
| Bamlanivimab | AbCellera Biologics/ Eli Lilly | 32 | >9,000 | >10,000 | [1,2] |
| Cilgavimab | Astra Zeneca | 18 | 62 | 1,079 | [1,2] |
| Tixagevimab | Astra Zeneca | 47 | 20 | 3,490 | [1,2] |
| P4A2 | DBT-THSTI, Govt. of India | 39 | 39 | 42 | This Study |

The numbers represent IC50 in ng/mL.

**References**

1. Aggarwal A, Stella AO, Walker G, Akerman A, Milogiannakis V, Brilot F, et al. SARS-CoV-2 Omicron: evasion of potent humoral responses and resistance to clinical immunotherapeutics relative to viral variants of concern. : 16.

2. D P, N S, P M, F G-B, C P, J B, et al. Considerable escape of SARS-CoV-2 Omicron to antibody neutralization. Nature. 2022;602. doi:10.1038/s41586-021-04389-z
