# Supplementary material for: A broadly neutralizing monoclonal antibody overcomes the mutational landscape of emerging SARS-CoV-2 variants of concern
Source: PLoS Pathog. 2022 Dec 12;18(12):e1010994. doi: 10.1371/journal.ppat.1010994 (PMC9779650; doi:10.1371/journal.ppat.1010994)
Supplement: S2 Table — (DOCX) [file ppat.1010994.s013.docx]

**Table S2.** Comparative epitope analysis of P4A2 mAb with other known ACE2 inhibiting antibodies

| **mAb^#^** | **Binding site with source** | **Reference** | **Remark** |
| --- | --- | --- | --- |
| P4A2 | 455-456, 475-477, 483-484 and 485-489 | This study | 455, 456, 475, 486, 487 and 489 are involved in ACE2 binding |
| S2X324 | 439-446, 498-502 and 506 (from paper) | [1] | No overlap with ACE2 binding site |
| LY-CoV1404 | 346 and 439-450 (7MMO) | [2] | 446 and 449 bind are involved in ACE2 binding |
| XGv051 | 453, 455, 475, 484, 489, 496, 498 and 501(from paper) | [3] | 453, 455, 475, 489, 496, 498 and 501 are involved in ACE2 binding |
| XGv264 | 439, 440, 445, 499 and 500 (from paper) | [3] | 500 is involved in ACE2 binding |
| XGv286 | 498, 499, 500 and 506 (from paper) | [3] | 498 and 500 are involved in ACE2 binding |
| S2K146 | 449, 452, 455, 484-502 and 505 (7TAS) | [4] | F486 present on a surface cavity while in P4A2 it is buried deep inside a hydrophobic pocket (452, 455, 486, 487, 489, 493, 496, 498, 500, 501, 502 and 505 are involved in ACE2 binding) |
| ZWD12 | 403, 446-449, 486 and 493-505 (7WWL) | [5] | F486 is on the surface of the paratope and the primary epitope is different from P4A2 (446, 449, 486, 493, 496, 498, 500, 501, 502 and 505 are involved in ACE2 binding) |
| 510A5 | 345, 346, 417, 439, 440, 449, 453, 456, 483-500 and 505 (7ws1) | [6] | F486 is on the surface of the paratope and the primary epitope is different from P4A2 (417, 449, 453, 456, 486, 487, 489, 493, 496, 498, 500 and 505 are involved in ACE2 binding) |
| 87G7 | 417, 421, 455-456, 484-489 and 493 (7r40) | [7] | Number of van der Waal and polar interactions are less than P4A2 (455, 456, 486, 487, 489 and 493 are involved in ACE2 binding) |
| JMB2002^*^ | 323-324, 532-538, 554-559 and 582-584 (7wka) | [8] | No overlap with ACE2 binding site |
| CAB-A17 | 403, 415, 417, 420, 421, 455, 460, 473, 475, 487, 489, 496, 501 and 505 (from paper) | [9] | 455, 475, 487, 489, 496, 501 and 505 are involved in ACE2 binding |
| B8-dIgA2 | 445, 446, 449, 484, 487, 489, 490, 493, 494 and 500 (from paper) | [10]*l* | 446, 449, 487, 489, 493 and 500 are involved in ACE2 binding |
| S3H3^*^ | 532-537, 554-556 and 581-584 (7WK8) | [11] | No overlap with ACE2 binding site |
| ABP-310 | 378, 408 and 414 (from paper) | [12] | No overlap with ACE2 binding site |
| NCV2SG48 | 449, 453-460, 473-477, 484-490 and 493-505 (from paper) | [13] | 449, 453, 455, 456, 475, 486, 487, 489, 493, 496, 498, 500, 501, 502 and 505 are involved in ACE2 binding |
| NCV2SG53 | 445-446, 449, 452, 455-456 and 475-501 (from paper) | [13] | 446, 449, 455, 456, 475, 486, 487, 489, 493, 496, 498, 500 and 501 are involved in ACE2 binding |
| VacW-209 | 371, 379, 405, 408, 414 and 415 (from paper) | [14] | No overlap with ACE2 binding site |
| S2E12 | 475, 476, 484, 485 and 486 (7K4N) | [7] | 475 and 486 are involved in ACE2 binding but the number of interactions are lower than P4A2 |
| COV2-2196 | 455, 456, 458, 475-480, 483-489 and 493 (from paper) | [15] | 455, 456, 475, 486, 487, 489, 493 are involved in ACE2 binding |
| S309 | 334-346 and 356-361 (6WPS) | [16] | No overlap with ACE2 binding site |

^#^These mAbs neutralise SARS-CoV-2 variants Alpha, Beta, Kappa, Delta and BA.1**.**

^*^Epitopes are not part of the RBD (330-520).

**References**

1. Park Y-J, Pinto D, Walls AC, Liu Z, De Marco A, Benigni F, et al. Imprinted antibody responses against SARS-CoV-2 Omicron sublineages. bioRxiv. 2022; 2022.05.08.491108. doi:10.1101/2022.05.08.491108

2. Westendorf K, Žentelis S, Wang L, Foster D, Vaillancourt P, Wiggin M, et al. LY-CoV1404 (bebtelovimab) potently neutralizes SARS-CoV-2 variants. bioRxiv. 2022; 2021.04.30.442182. doi:10.1101/2021.04.30.442182

3. Wang L, Fu W, Bao L, Jia Z, Zhang Y, Zhou Y, et al. Selection and structural bases of potent broadly neutralizing antibodies from 3-dose vaccinees that are highly effective against diverse SARS-CoV-2 variants, including Omicron sublineages. Cell Res. 2022;32: 691–694. doi:10.1038/s41422-022-00677-z

4. Park Y-J, De Marco A, Starr TN, Liu Z, Pinto D, Walls AC, et al. Antibody-mediated broad sarbecovirus neutralization through ACE2 molecular mimicry. bioRxiv. 2021; 2021.10.13.464254. doi:10.1101/2021.10.13.464254

5. Chi X, Yan R, Zhang J, Zhang G, Zhang Y, Hao M, et al. A neutralizing human antibody binds to the N-terminal domain of the Spike protein of SARS-CoV-2. Science. 2020;369: 650–655. doi:10.1126/science.abc6952

6. Guo H, Gao Y, Li T, Li T, Lu Y, Zheng L, et al. Structures of Omicron spike complexes and implications for neutralizing antibody development. Cell Reports. 2022;39: 110770. doi:10.1016/j.celrep.2022.110770

7. Du W, Hurdiss DL, Drabek D, Mykytyn AZ, Kaiser FK, González-Hernández M, et al. An ACE2-blocking antibody confers broad neutralization and protection against Omicron and other SARS-CoV-2 variants of concern. Sci Immunol. 2022;7: eabp9312. doi:10.1126/sciimmunol.abp9312

8. Yin W, Xu Y, Xu P, Cao X, Wu C, Gu C, et al. Structures of the Omicron spike trimer with ACE2 and an anti-Omicron antibody. Science. 2022;375: 1048–1053. doi:10.1126/science.abn8863

9. Sheward DJ, Pushparaj P, Das H, Kim C, Kim S, Hanke L, et al. Structural basis of Omicron neutralization by affinity-matured public antibodies. bioRxiv; 2022. p. 2022.01.03.474825. doi:10.1101/2022.01.03.474825

10. Zhou B, Zhou R, Chan JF-W, Zeng J, Zhang Q, Yuan S, et al. SARS-CoV-2 hijacks neutralizing dimeric IgA for enhanced nasal infection and injury. bioRxiv; 2021. p. 2021.10.05.463282. doi:10.1101/2021.10.05.463282

11. Hong Q, Han W, Li J, Xu S, Wang Y, Xu C, et al. Molecular basis of receptor binding and antibody neutralization of Omicron. Nature. 2022;604: 546–552. doi:10.1038/s41586-022-04581-9

12. Pelzek AJ, Ebtehaj S, Lulo J, Zhang L, Balduf O, Dolan L, et al. A potent SARS-CoV-2 neutralizing antibody recognizing a conserved epitope with broad mutant variant and SARS-CoV activity. bioRxiv; 2022. p. 2022.02.06.479332. doi:10.1101/2022.02.06.479332

13. Germinal center-derived broadly neutralizing antibodies adapt to SARS-CoV-2 antigenic drift | bioRxiv. [cited 23 Oct 2022]. Available: https://www.biorxiv.org/content/10.1101/2022.01.26.477937v1.full

14. Ju B, Zheng Q, Guo H, Fan Q, Li T, Song S, et al. Immune escape by SARS-CoV-2 Omicron variant and structural basis of its effective neutralization by a broad neutralizing human antibody VacW-209. Cell Res. 2022;32: 491–494. doi:10.1038/s41422-022-00638-6

15. Dong H, Su A, Lv D, Ma L, Dong J, Guo N, et al. Correction to Development of Whole-Porcine Monoclonal Antibodies with Potent Neutralization Activity against Classical Swine Fever Virus from Single B Cells. ACS Synth Biol. 2020;9: 978. doi:10.1021/acssynbio.0c00160

16. Pinto D, Park Y-J, Beltramello M, Walls AC, Tortorici MA, Bianchi S, et al. Cross-neutralization of SARS-CoV-2 by a human monoclonal SARS-CoV antibody. Nature. 2020. doi:10.1038/s41586-020-2349-y
