# Supplementary material for: A broadly neutralizing monoclonal antibody overcomes the mutational landscape of emerging SARS-CoV-2 variants of concern
Source: PLoS Pathog. 2022 Dec 12;18(12):e1010994. doi: 10.1371/journal.ppat.1010994 (PMC9779650; doi:10.1371/journal.ppat.1010994)
Supplement: S3 Table — (PDF) [file ppat.1010994.s014.pdf]

Table S3. Crystallographic data and refinement statistics for P4A2 Fab:Spike-RBD complex

**Data collection statistics**	
Wavelength (Å)	0.97926
Space Group	$P3_12_1$
Cell Constants (Å)	85.3 85.3 205.2
Resolution (Å)	3.0 Å (3.24-3.0)[a]
R_{merge}[b]	9.4 (69.6)
I/σI	7.3 (2.1)
Completeness (%)	100 (100)
Redundancy	9.1 (9.1)
CC(1/2)	0.975 (0.79)
Refinement	
Resolution (Å)	73.88-3.0 Å
No. of Reflections	17972
R_{work}[c]/R_{free}	23.0/28.2
No. of atoms	
Protein	4567
Water	92
R. M. S. deviations	
Bond lengths (Å)	0.004
Bond angles (°)	0.722

[a]Values in parentheses are for the highest resolution shell.

[b]$R_{merge} = \Sigma|I - \langle I \rangle|/\Sigma I$, where I is the integrated intensity of a given reflection.

[c]$R_{work} = \Sigma||F_{obs}| - |F_{calc}||/\Sigma|F_{obs}|$. R_{free} was calculated using 7% of data excluded from refinement.
