# Supplementary material for: A broadly neutralizing monoclonal antibody overcomes the mutational landscape of emerging SARS-CoV-2 variants of concern
Source: PLoS Pathog. 2022 Dec 12;18(12):e1010994. doi: 10.1371/journal.ppat.1010994 (PMC9779650; doi:10.1371/journal.ppat.1010994)
Supplement: Attachment — Submitted filename: 126312_1_rebuttal_2407621_r4v3nw.docx [file ppat.1010994.s015.docx]

**Manuscript number:** RC-2022-01423

**Corresponding author(s):** Rajesh, Kumar

*[Please use this template only if the submitted manuscript should be considered by the affiliate journal as a full revision in response to the points raised by the reviewers.*

**Response to Reviewer’s Comment**

We are very thankful to the members of the editorial board for providing us with an opportunity to resubmit a revised version of our manuscript (manuscript number Review Commons Refereed Preprint #RC-2022-01423). The authors also appreciate the effort taken by the reviewers to provide comprehensive input on the manuscript. An effort from the authors was made to edit the entire manuscript to address the specific query raised. We have enlisted here the responses to the relevant queries sequentially.

**Reviewer #1**: ***Evidence, reproducibility and clarity (Required)***

***Summary:*** *The authors generated a panel of anti-SARS-CoV-2 mAbs using mouse hybridoma technology, by immunizing BALB/c mice with purified RBD protein that corresponds to Alpha variants of SARS-CoV-2 (N501Y). These screening activities gave rise to a monoclonal antibody called P4A2, which demonstrated exceptional efficient and broad neutralization of both ancestral SARS-CoV-2 as well as a number of variants of concern (VOCs), which, importantly, also includes the Omicron VOC. The authors solved a crystal structure of the Fab of P4A2 in complex of the RBD, showing that it binds to a region that is not affected by mutations in VOCs. Further, the structures show that P4A2 binding blocks the RBD-Ace2 interaction; this is the most common mechanism by which antibodies and other biomolecules neutralize SARS-CoV-2. The paper includes also biochemical analyses to determine affinities against RBDs of different VOCs and a demonstration of the potency of P4A2 to treat infected K18-hACE2 transgenic mice.*

1. **Comment:** *Overall, the data looks sound and credible. In particular, the data on the neutralization of Omicron VOC make sense, as the binding epitope on the RBD recognized by P4A2 is conserved in relevant VOCs.*

**Response:** The authors are overwhelmed with the appreciative and positive feedback from the reviewer. We want to inform the reviewer that the data associated with all experimental procedures for characterization of P4A2 clone has been repeated three times and is reproducible.

2. **Comment:** *The in vivo experiments were performed at an extensive scale. Also, it is impressive how potent P4A2 is vis-à-vis other marketed monoclonal antibodies against Omicron (Figure S8). Further experiments as such are not needed to support the claims of the study.*

**Response:** We appreciate the keen insights of reviewer on the positive feed back

3. **Comment:** *Figure 1 and elsewhere in main text and other figures: I would strongly recommend to also provide molarities and not only weigth/volume values to describe the neutralization efficiencies.*

**Response:** We have amended the Figure 1 and text where required as per reviewer’s suggestion.

4. **Comment:** *The sentence: "The residues of RBD, that interact with the paratope, are part of the RBM involved in interacting with residues of human ACE2." is rather complicated. The residues of RBD, that interact with the paratope, are called the epitope...I suggest to simplify.*

**Response:** As suggested by the reviewer, we have changed the sentence to “The residues that constitute the epitope on RBD, are involved in interacting with residues of human ACE2.” Also we modified an earlier sentence in the text to define the epitope and it now reads as “The residues of the RBD that interact with the paratope of the P4A2 Fab and constitute the epitope are 455Leu, 456Phe, 483Val, 484Glu, 485Gly, 486Phe, 487Asn, and 489Tyr (Fig. 1D).”

**Reviewer #1 (Significance (Required)):**

5. **Comment:** *The main novel finding is the fact that they authors found an antibody with potent neutralization efficiency against all relevant VOCs, including Omicron. The structural, biochemical, and cell biology/in vivo work solidly support this central claim.*

*Other than that, the study does not offer much novelty in terms of technological advance or biological insights. This is, however, not surprising, because there were so many SARS-CoV-2 antibody studies published in the last two years.*

**Response**: There are large number of SARS-CoV-2 antibody studies but the P4A2 antibody described in this manuscript can neutralize all known VoCs of SARS-CoV-2. Since the P4A2 epitope overlaps with the ACE2 binding site, mutations in Spike-RBD that abrogate P4A2 binding will also reduce Spike-RBD:ACE2 interaction. Consequently, P4A2 may be effective against new VoCs of SARS-CoV-2 that appear in the future.

*Elements that might have added additional values would have been the*

i) *generation/analysis of escape mutations (since P4A2 addresses only one main epitope, I would expect that the virus can escape fast, but maybe, there is a strong trade-off between Ace2 recognition and antibody escape?)*

***Response:*** Maher et al; predict that the following mutations will perturb the ability of therapeutic mAbs to recognize Spike-RBD: A344S, R346K, K417T/N, K444N, G446V, L452R/Q, L455F, G476S, S477I, T478K, V483F, E484K/Q, F490S, S494L/P. These mutations are predicted to adversely affect the activity of sotrovimab, letesevimab, imdevimab, bamlanivimab, casirivimab and CT-P59. However, we observe that none of these mutations will lead to the loss of stabilizing interactions or will give rise to steric clashes at the P4A2 Fab:RBD interface. As a result, these mutations in the RBD will not reduce the ability of P4A2 to neutralize the SARS-CoV-2. We have included a supplementary figure in the manuscript that depicts that P4A2 binding is unaffected by any of these predicated mutations. So believe that P4A2 will be effective against the future emerging SARS-CoV-2 variants. Also, as mentioned by the reviewer, since there is substantial overlap between the Spike-RBD epitope for P4A2 and the residues that are critical for binding ACE2, mutations in Spike-RBD will reduce the affinity of P4A2.

The epitopes comparison analysis of P4A2 with other ACE2 inhibiting antibodies shows that 87G7, 510A5, S2X324, XGv051, XGv264, XGV286, ZWD12 showed some overlap with that of P4A2 but only the P4A2 mAb possesses a hydrophobic cleft into which the 486Phe residue is buried. Based on available information, the P4A2 forms multiple interactions with its cognate epitope on Spike-RBD, and multiple residues present in this epitope are critical for interaction with ACE2 (Fig, S11 and Table 2). will also adversely impact ACE2 recognition.

**Supplementary Table 2. Comparative epitope analysis of P4A2 mAb with other known ACE2 inhibiting antibodies**

| **mAb** | **Neutralizing variants** | **Binding site with source** | **Author** | **Remark** |
| --- | --- | --- | --- | --- |
| **P4A2** | All | 455-456, 475-477, 483-484, and 485-489 | Parray et al | 455, 456, 475, 486, 487, and 489 are involved in ACE2 binding |
| **S2X324** | All | 439-446, 498-502, 506 (from paper) | Young-Jun-Park et al | No overlap with ACE2 binding site |
| **LY-CoV1404** | All | 346, 439-450 (7MMO) | Kathryn Westendorf et al | 446 and 449 bind are involved in ACE2 binding |
| **XGv051** | All | 453,455,475,484,489,496,498,501(from paper) | Lei Wang et al | 453, 455, 475, 489, 496, 498 and 501 are involved in ACE2 binding |
| **XGv264** | All | 439,440,445, 499,500(from paper) | Lei Wang et al | 500 is involved in ACE2 binding |
| **XGv286** | All | 498,499,500,506 (from paper) | Lei Wang et al | 498 and 500 are involved in ACE2 binding |
| **S2K146** | All | 449, 452, 455, 484-502, 505 (7T, resioAS) | Park YJ et al | F486 present on a surface cavity while in P4A2 it is buried deep inside a hydrophobic pocket (452, 455, 486, 487, 489, 493, 496, 498, 500, 501, 502, 505 are involved in ACE2 binding) |
| **ZWD12** | All | 403, 446-449, 486, 493-505 (7WWL) | Xiangyang Chi et al | F486 is on the surface of the paratope and the primary epitope is different from P4A2 (446, 449, 486, 493, 496, 498, 500, 501, 502 and 505 are involved in ACE2 binding) |
| **510A5** | All | 345, 346, 417, 439, 440, 449, 453, 456, 483-500, 505 (7ws1) | Hangtian Guo et al | F486 is on the surface of the paratope and the primary epitope is different from P4A2. (417, 449, 453, 456, 486, 487, 489, 493, 496, 498, 500, 505 are involved in ACE2 binding) |
| **87G7** | All | 417, 421, 455-456, 484-489, 493 (7r40) | Wenjuan Du et al | Number of van der Waal and polar interactions are less than P4A2 (455, 456, 486, 487, 489, 493 are involved in ACE2 binding) |
| **JMB2002*** | All | 323-324, 532-538, 554-559, 582-584 (7wka) | Wanchao Yin et al | No overlap with ACE2 binding site |
| **CAB-A17** | All | 403,415,417,420,421,455,460,473,475,487,489,496,501,505(from paper) | Daniel J. Sheward et al | 455, 475, 487, 489, 496, 501 and 505 are involved in ACE2 binding |
| **B8-dIgA2** | All | 445,446,449,484,487,489,490,493,494,500 (from paper) | Biao Zhou et al | 446, 449, 487, 489, 493, 500 are involved in ACE2 binding |
| **S3H3*** | All | 532-537, 554-556, 581-584 (7WK8) | Qin Hong et al | No overlap with ACE2 binding site |
| **ABP-310** | All | 378,408,414 (from paper) | Adam J. Pelzek et al | No overlap with ACE2 binding site |
| **NCV2SG48** | All | 449, 453-460, 473-477, 484-490, 493-505 (from paper) | Kiyomi Shitaoka et al | 449, 453, 455, 456, 475, 486, 487, 489, 493, 496, 498, 500, 501, 502 and 505 are involved in ACE2 binding. |
| **NCV2SG53** | All | 445-446, 449, 452, 455-456, 475-501 (from paper) | Kiyomi Shitaoka et al | 446, 449, 455, 456, 475, 486, 487, 489, 493, 496, 498, 500, and 501 are involved in ACE2 binding. |
| **VacW-209** | All | 371,379,405,408,414,415(from paper) | Bin Ju et al | No overlap with ACE2 binding site |
| **S2E12** | All | 475,476,484,485,486 (7K4N) | Wenjuan Du et al | 475 and 486 are involved in ACE2 binding but the number of interactions are lower than P4A2 |
| **COV2-2196** | All | 455,456,458,475-480,483-489,493 (from paper) | Jinhui Dong et al | 455, 456, 475, 486, 487, 489, 493 are involved in ACE2 binding. |

*Epitopes are not part of the RBD (330-520).

ii) *A cryo-EM structure of the Spike/Fab complex (maybe, the antibody might have changed the dynamic equilibrium of up/down conformations of the RBDs?)*

***Response:*** We have used computational tools to generate structural models of the P4A2 Fab bound to the Spike RBD. Since there are at least 200 different structures of Spike trimer bound to different Fab molecules, and we have the crystal structure of the Spike-RBD in complex with P4A2 Fab, we can use computational tools such as structural alignment, docking and energy minimization to obtain robust models of the Spike trimer bound to P4A2 Fab. The analysis clearly shows that, for both the “up” as well as “down” conformation, the P4A2 Fab can bind to the Spike trimer. Since the ACE2 molecule can only bind to the Spike-RBD when it is in the “up” conformation, P4A2 binding will effectively prevent engagement with the ACE2 receptor to prevent cellular entry. We have shown that P4A2 mAb can neutralize all known VoCs of SARS-CoV-2 in both cell culture and animal models of infection and this is due to its ability to bind to a region on the Spike-RBD that is important for ACE2 binding. Consequently, CryoEM structure of P4A2 Fab bound to Spike trimer will not add substantial new information to the study presented in the manuscript.

*I say "might have added", because such experiments had been reported as well so many times.*

*Having said this, I consider this paper a solid piece of work that appeals to researchers working on antibodies and related biomolecules to broadly inhibit VOCs of SARS-CoV-2.* *My own expertise lies in nanobodies, display technologies and structural biology*

**Response:** Authors appreciate the critical analysis related to the novelty of the P4A2 antibody. The major significance related to this antibody is that it is natural and potential neutralizing which we have demonstrated as a proof of concept. We agree with the reviewer comment there are broadly neutralizing antibodies that are effective against all VOCs. Here we report an bnAb that recognizes an epitope on the RBM of RBD and its unique features enable it to overcome mutations found in the VOCs. The discovery and comprehensive structural analysis of P4A2 provide valuable insight for broad and potent neutralization of SARS-CoV-2.

**Reviewer #2 (Evidence, reproducibility and clarity (Required))**:

**Summary:** *In this manuscript, the authors reported the development of mouse monoclonal antibodies (mAbs) against a receptor-binding domain (RBD) of SARS-CoV-2 Alpha variant. One of the obtained mAbs, designated P4A2, showed KD < 1 pM activity against Alpha, Beta, and Delta variants, and sub-nM KD values against Gamma, Kappa, and variants (no KD value against Omicron was shown in the text). This mAb also inhibits the ACE2-RBD interaction, thus exhibiting neutralization activity against the above variants. The X-ray crystal structure of the mAb Fab/Alpha RBD, the computational models against other variants, and the closed form of the trimer explained the high affinity and neutralization activity. Moreover, the authors tested the ability of the P4A2 mAb to neutralize SARS-CoV-2 WA1, Kappa, Beta, and Delta variants, in order to demonstrate the therapeutic potential of this mAb.*

1. **Comment:** *KD values presented against some variants are very low (KD < 1 pM), but the data were obtained at 3.7 nM to 300 nM concentrations. Are these extremely low values actually reliable?*

**Response:** We agree with the reviewer that the KD value against some variants are very low (KD < 1 pM), even at the concentration range used by us in the experiment. Since there is no dissociation in such a case the KD read out will be very high in the pM range as seen here. Our acquired data plot shows that P4A2 binding of analyte is very strong which does not dissociate over the dissociation time range used in the experimental condition, which could signify the very high affinity, as per the current BLI technology can't measure very low affinity in the pM range.

However, at the same time with the same concentration range, the experiment has successfully calculated the affinity of other VOC’s (Beta, Gamma, Kappa and Omicron) in sub nano molar range where the mAb dissociation (Kdis) was factually observed. Hence, we believe that calculated affinity though very high is with confidence reflects the affinity of P4A2 is in low picomolar range for rest VOC’s

**Comment:** *P4A2 mAb against Omicron RBD has nanomolar affinity according to Figure 1K and the main text. This is inconsistent with the very low IC50 in Figure S8 (42 ng/mL corresponds to* *0.2 nM). Explain the reasons why the IC50 value was significantly lower than the KD value in the main text.*

**Response:** The authors acknowledge the interest of the reviewer towards the potency of the mAb. Binding affinity and functional neutralization assays are two different experiments performed by us. In BLI we have calculated the binding affinity of P4A2 mAb with RBD, where we believe that one antibody binds with one RBD molecule (1:1) but in live virus based functional assay we are talking in terms of whole virus, where one antibody molecule has the spatial freedom to interact with probably more than one protomers on the spike trimers In addition to that other probability is that one antibody molecule can engage spike protein of two different viral particles. Consequently, there will be a greater effect on neutralization than can be expected from BLI experiments. Also we here emphasis on the fact that the IC50 calculated by live virus neutralization are more realistic in terms of functional assay.

2. **Comment:** *Figure 1J shows significant drops in activity against Beta (IC50 = 5 nM?) and Gamma (IC50 = 2 nM?) RBDs. Even the affinities were retained as sub-nM KD. Explain the reasons for this in the main text.*

**Response:** The affinity of P4A2 is for Spike-RBD from the beta, gamma, delta, kappa and omicron variants is in the nanomolar or sub-nanomolar range. For the beta and gamma variants, the affinity is in the nanomolar range as compared to other VoCs. It is possible that mutation of the E484 residue maybe responsible for this. From the crystal structure of Spike-RBD:P4A2 Fab complex, it is clear that this residue forms interactions with the Fab paratope through the polypeptide backbone atoms. The side chain is not involved in these interactions, but the change in electrostatic surface of the molecule due to these mutations may increase the Koff leading to a decrease in the equilibrium affinity. However, the affinity is still in the nanomolar range and therefore the E484 mutations have minimal effect the ability of P4A2 to neutralize the SARS-CoV-2 virus in cell culture and animal models. We have elaborated the same in the main text .

**Comment:** *"Kon" and "Koff" should be changed to "kon" and "koff".*

**Response:** The manuscript has been amended with the suggested changes.

3. **Comment:** *Fix Figure 1D. A copy of Figure 1G has overlapped it.*

**Response:** Necessary changes have been incorporated in the manuscript.

4. **Comment:** *Show the measured binding parameters of P4A2 against Omicron in Figure 1K.*

**Response:** The Figure 1K has been modified as per reviewers’ suggestion.

5. **Comment:** *Show the measured IC50 in Figure 1J*

**Response:** IC50 values have been incorporated in 1J

6. **Comment:** *Figure S8 was not reffered in the main text.*

**Response:** We appreciate reviewer’s sharp observations and are apologetic for missing out the Figure S8. The changes have been incorporated in the manuscript as recommended

7. **Comment:** *The graphics for Beta and Omicron in Figure S3 are not clear.*

**Response:** The Quality for Figure S3 have been enhanced as per recommendation

8. **Comment:** *I believe the labels in Figure S4 are incorrect. There is data for Delta in Figure S4A.*

**Response:** We appreciate reviewer’s remarks, the correction has been made in revised manuscript.

**Reviewer #2 (Significance (Required)):**

11. **Comment:** *The significance of this paper is the demonstration of the broad-range affinity and neutralization activity of the mouse P4A2 antibody against various VOCs, as well as the X-ray structure of the antibody and the RBD of the Alpha variant. While the solved structure is of interest, similar broad-range antibodies were already published on (e.g., Science 2022, 375(6582); Sci Immunol. 2022, eabp9312; Signal Transduction and Targeted Therapy 2022, 7(43)). Moreover, in order to use the antibody toward therapeutic purposes, the authors would need to humanize the antibody to avoid an undesired immune response since the studied antibody is derived from a mouse. These drawbacks will limit the audience of the paper significantly.*

**Response:** Authors agree with the reviewers’ viewpoint associated with significance of antibody with the current data. We would also like to draw attention of the reviewer towards the development procedure, that this is a natural antibody with other robust biological characteristics and has been isolated and a proof of concept has been given for its therapeutic potential in cell culture and animal models. As many of the commercially viable antibodies are derived from mouse hybridoma and subsequently humanized use in therapy. Thus, humanization of the murine mAbs is a standard procedure and the P4A2 mAb has been already licensed to an industrial partner for generating the humanized version.

Regarding the reviewers point about similar studies with reference to the three papers, our study is different from these in the following ways:

1. Science 2022, 375(6582): The antibody in this study JMB2002 does not neutralize the delta strain of SARS-CoV-2 but the P4A2 mAb described in our manuscript is able to neutralize all known VoCs of SARS-CoV-2.
2. Sci Immunol. 2022, eabp9312: Compared to the 87G7 mAb described in this study, the P4A2 epitope shows greater overlap with the ACE2 binding site. Also, in this study the ability of 87G7to neutralize the kappa strain was not tested.
3. Signal Transduction and Targeted Therapy 2022, 7(43): The 3E8 mAb mentioned in this study actually binds the ACE2 molecule and is able to prevent infection by the beta, gamma, delta and omicron strains of SARS-CoV-2. There is no structural data in this manuscript and therefore the mechanism of virus neutralization is not clear. Also, because it binds to the ACE2 receptor and not to the spike-RBD, its utility as an effective therapeutic for SARS-CoV-2 may be limited due to side effects.

**Reviewer #3 (Evidence, reproducibility and clarity (Required)):**

**Summary:** *Parray et al. describe the generation and characterization of a mouse monoclonal antibody capable of neutralizing several variants of SARS-CoV-2, including the recently emerged and highly infectious omicron variant. The antibody was obtained by immunizing mice, and tested in an in vivo protection model as well as by in vitro neutralization assays. Results of these experiments suggested that the antibody was capable of neutralizing variants other than the one against which it was raised, and to elucidate the structural basis for this broad specificity, the structure of the antibody Fab/ spike protein receptor binding domain complex was also studied by X-ray crystallography and computational modelling in order to investigate the nature of the interaction between the antibody and its antigen. The methods are suitable for the purpose, and in general well described. I have a number of comments, mostly minor in nature.*

1. **Comments:** *Fig 1I shows the critical result that the IC50 against omicron is similar to that against delta. The horizontal axis of this figure is not very clear, i.e., the relationship between the numbers and the data points is ambiguous. From what I can see, it looks as though the delta was tested at a lower concentration than the omicron, which in the context of a direct comparison seems like not the best design. Also, the reported IC50 of 42 or 39 ng/ml are right at the bottom of the range of the values tested. Possibly the authors were not expecting that the P4A2 antibody would perform so well, but since it appears to do so, wouldn't it be worth testing down to a concentration of antibody at which the neutralization disappears? This would enable the calculation of a more reliable IC50, I propose. Since the authors contend that a major aspect of the significance of the report is the fact that antibody P4A2 is more potent than sotrovimab, I would contend that an accurate and reliable measurement of this parameter is of paramount importance.*

**Response:** Authors appreciate reviewers’ recommendations and the data representation has been accordingly incorporated in the revised manuscript. We have repeated the experiment and represented the new figure data in the revised manuscript. As per reviewers suggestion we have also compared binding affinity and neutralization potential of P4A2 and sotrovimab, data for the same has been added in the revised manuscript.

2. **Comments:** *In Figure 1K the concentrations could informatively be displayed on the figure or at the very least in the legend. Wouldn't it be a good idea to state the calculated Kd somewhere?*
3. **Response:** The K_D_ values have been incorporated in the amended manuscript as per reviwers’ suggestion.
4. **Comment:** *Eyeballing Figure 2B it looks as though the IC50 is something like 500 ng/ml, i.e., 10 times higher for the than for non-immunized variants. Which variant was tested here? I understand that this is a different assay, but is this a reliable finding? Concretely, how many times were each of these assays repeated? Many journals have a policy that this this information should be specified, and I think it would be helpful here. According to the Methods section, if I understand correctly, II62 and CR3022 mAb against SARS-CoV-2 were used as controls in this experiment. Please can we see them plotted?*

**Response:** Authors acknowledge the Reviewers’ apprehension regarding the IC50 values in Figure 2B and reproducibility of associated data. We would like to inform the Reviewer the experiments have been repeated 3 times. t (A statement stating that has been incorporated in the manuscript text). Also the desirable plots for the control antibodies have been inserted in the revised manuscript as per suggestion.

5. **Comment:** *Figure 2B is labelled hku1 but the text describes results for hku4.*

**Response:** Correction done in the revised manuscript.

6. **Comment:** *Please provide sequences or accession numbers for the full-length spike proteins of SARS-CoV-1, SARS-CoV-2, MERS, HKU1, OC43, NL63,and 229E.*

**Response:** All of the constructs were codon optimized. The constructs were a generous gift from the author from the manuscript “Cross-reactive serum and memory B-cell responses to spike protein in SARS-CoV-2 and endemic coronavirus infection” Ge Song et al. Nat Commun. 2021.Nat Commun. 2021 May 19;12(1):2938.doi: 10.1038/s41467-021-23074-3.PMID: 34011939. The detail regarding that has also been mentioned in the material and method section “sub section: Viruses and antibodies

7. **Comment:** *Figures 2C and 2E vertical axis looks like body mass rather than change. The keys are hard to understand because the lines too short to see their line type.*

**Response:** The Y-aixs in figure 2C and 2E is correctly labelled and represents percentage change in the body mass which was calculated as a percentage change in the body mass of that animal as compared to the body mass recorded on day zero.

For better understanding of readers, we have provided a supplementary figure that clearly depicts the differences in change in body mass at day 6 (Fig. S5A).

8. **Comment:** *The precision and accuracy of the timelines in Figure S5A should be improved. For example, in the therapeutic context, it looks as though the antibody was administered at 1 day post infection, but the text suggests 6-12 hours. In this context, 12 hours is significantly different from 6, and the actual time should be made clear. My suggestion would be to remove this Supplementary figure and use arrows in Figures 2E and 2C to show the time points of virus and antibody administration. The actual times would be more helpful than a range. If the time interval different between experiments between 6 and 12 hours, then the results should be shown separately.*

**Response:** Authors acknowledge the reviewers’ suggestions and the recommended change are incorporated for the timelines in the manuscript. Initial standardization therapeutic animal experiments was done at two time point 6h and 12 h with SARS-CoV2. We did not find any significant difference in protection efficacy at both the time points, so for the other VOCs protection studies we used 12h infusion time. We have corrected our statements and incorporated the data collected for 12 h infusion for all VOCs. As per reviewer suggestion we have removed the figure 5SA and the actual time should be made clear in figure, figure legend and text of the manuscript.

9. **Comment:** *I couldn't find the sequence of antibody P4A2 anywhere, or a reference to a public database containing it.*

**Response:** As per reviewer suggestion we have added variable heavy and light chain gene sequence of P4A2 in the supplementary figure **(Fig 510A)**. Provisional PDB for RBD-P4A2 Fab has been submitted in database and it will be available in the public domain after acceptance of the manuscript.

10. **Comment:** *In the Methods section, the description "The purified Fab and RBD proteins were mixed at 1:1.7 ratio" should make clear whether this is by mass or molarity (or whatever).*

**Response:**

The proteins were mixed in the molar ratio of 1:1.7. The method section has been amended in the revised manuscript to include this information.

11. **Comment:** *In my pdf, in Figure S3, the images for "omicron" are not obviously different from the "negative" images. Is this just image quality? If so, I suggest replacing with higher quality images. If the labling of omicron infected cells is really so different from cells infected with other variants, this should be explained.*

**Response:** The quality for the Figure S3 has been improved as suggested by the reviewer.

12. **Comment:** *In fig 2F, I guess that for example 10^6 should be 6, and so on, if the scale shows log10 (copy number).*

**Response:** The Y axis of Figure 2F has been modified in accordance to the recommendations.

13. **Comment:** *In Figure S4 the labeling seems to have been displaced.*

**Response:** Labelling has been corrected in the revised manuscript.

**Reviewer #3 (Significance (Required)):**

13. **Comment:** *The significance of this report include the isolation of an antibody capable of broad neutralization of multiple SARS-CoV-2, a finding of obvious medical significance, and the investigation of its structural underpinnings. The authors compare the properties of their antibody to those of sotrovimab, and assert that theirs is more potent. According to Cameroni et al. (PMID: 35016195) Sotrovimab, which is a fully human monoclonal antibody and therefore arguably one step closer to usefulness in the clinic, and has been licensed for therapeutic use by major regulatory authorities, has an IC50 of 200 ng/ml or so. Antibody P4A2 described in this manuscript is reported to have a Kd some five times lower than this. If this can be confirmed (I mention some reasons in the Minor Comments why I do not yet consider this definitive), then this presumably represents a significant advance.*

**Response:** The authors are indebted to the Reviewers for inspiring feedback on the manuscript. We agree with the Reviewer that human antibodies are one step closer to usefulness as compared to mouse antibodies. We want to emphasis that in recent years advent in antibody engineering methodologies has made humanization process a standard methodology in the field. A number of companies has also started providing this service in a time frame of 6 to 8 weeks.

The epitope recognize by P4A2 is different from the Sotrovimab. We have compared the neutralization potential of both mAbs in the same assay and data clearly showed that P4A2 has an upper edge in terms of neutralization potential over Sotrovimab (Fig. S8). Structural data also shows that P4A2 due to its unique mode of binding has potential to neutralize the current circulating VOCs and will be highly effective against future variants as well.

We also compared the affinity of both mAbs with omicron RBD and we find that both antibodies binds with almost similar KD values.
