# Supplementary material for: A broadly neutralizing monoclonal antibody overcomes the mutational landscape of emerging SARS-CoV-2 variants of concern
Source: PLoS Pathog. 2022 Dec 12;18(12):e1010994. doi: 10.1371/journal.ppat.1010994 (PMC9779650; doi:10.1371/journal.ppat.1010994)
Supplement: Attachment — Submitted filename: Point by point responses 26102022.docx [file ppat.1010994.s016.docx]

**Response to Reviewers’ comments**

**Comment:** *All 3 reviewers stated that the authors satisfactorily addressed previous comments, and that this work is highly significant towards tackling SAR-CoV-2 variants. Reviewers 2 and 3 provided helpful comments to increase manuscript quality (both in terms of the text and figures). Thank you in advance for addressing each reviewer's comments.*

**Response:** We are very grateful to the Editorial Board for providing us with an opportunity to revise our manuscript (manuscript number PPATHOGENS-D-22-01266 - [EMID:92e891bf6841f6ac]). The authors also appreciate the effort taken by the reviewers to provide comprehensive input on the manuscript. An effort has been made by the authors to amend the entire manuscript to address the specific queries raised. We have enlisted here the responses to the relevant queries sequentially, as well as incorporated the suggestions in the edited manuscript.

**Reviewer #1**

**Part I –** ***Summary:*** *Strengths/weaknesses of study, novelty/significance, general execution and scholarship*.

1. **Comment:** *The candidate has taken a lot of effort to address the concerns of all the reviewers and to me, it is highly satisfactory. Maybe they can comment (in conclusion) more on new variants of covid and their potential to get neutralized by their MCA but this is extremely minor point.*

**Response:** The authors are overwhelmed with appreciative and positive feedback from the reviewer. We want to inform the reviewer that the comments on new variants of COVID and their possibilities for getting neutralized by the monoclonal antibody of the present research (i. e., P4A2) have been incorporated into the conclusion of the revised manuscript as per the Reviewer’s suggestion.

**Part II –** ***Major Issues:*** *Key Experiments Required for Acceptance*

2. **Comment:** *No major issues - accepted in the present form*

**Part III** – ***Minor Issues:*** *Editorial and Data Presentation Modifications*

3. **Comment:** *None*

**Reviewer #2**

**Part I –** ***Summary:*** *Strengths/weaknesses of study, novelty/significance, general execution and scholarship*.

1. **Comment:** *With one minor exception, the authors have responded fully and constructively to the comments I made at the initial review. The main thing I would like to see improved in the current version* *is the description of the methods for the focus reduction neutralization assay. This request is prompted by the new image of the omicron-infected cells in Figure S3. I had previously asked if a better image were available, and the authors have kindly provided one. The new image confirms the impression that the omicron variant produces fewer foci than the other variants. It would therefore make sense to describe the methods for the focus reduction neutralization assay in sufficient detail that a reader could judge whether the very different kinetics of the infection were handled in a way that meant that the IC50 for this variant and the others are comparable. Ideally**, images of the neutralization plates could be provided containing at least one series for each of the agents shown on the plot, along with a description of how the parameters of infection were measured and analyzed.*

**Response:** The authors acknowledge the interest of the Reviewer. The authors have elaborated on the description of the method for the focus reduction neutralization assay in the revised manuscript. A comprehensive description of the infection and analysis procedure has also been summarized in the amended manuscript. Also, a new **supplementary figure S8** has been added in accordance with the Reviewer’s suggestion on images of the neutralization plates could be provided containing at least one series for each of the agents shown on the plot, along with a description of how the parameters of infection were measured and analyzed.

2. **Comment:** *Also on the theme of comparability, I believe that the comparison shown in S8 between experimentally determined IC50 for P4A2, with values from the literature for other agents is of rather limited value presented as it stands, because of the very different values obtained by different labs with nominally similar assays. The experimental comparison* *between P4A2 and sotrovimab provided by the authors in S9 is much more informative. If these* *figures S8 and S9 could be combined, so that inter-laboratory variation in the IC50 measured for the same agent were immediately obvious, it would aid intepretation of both figures. S9 is such an important figure that I don't know why it was not included in the main figures, for example in Figure 1.*

**Response:** We appreciate the Reviewer’s remark on the theme of experimental comparability between P4A2 and sotrovimab data shown in the present manuscript (figures S8 and S9). The correction has been made in the revised manuscript as per the Reviewer’s suggestion. Figure S9 has been shifted in the main manuscript as Figure 3 and S8 figure has been shifted as supplementary Table S1.

3. **Comment:** *Regarding my previous comment about the label on the vertical axis of figures 2C and 2E, despite the authors, response, I stand by my initial opinion, and extend it to S5A. To me, a change in body mass of 100% means that the body mass increased or decreased by 100%, suggesting that on day 1 the animals doubled their weight, by day 2 had tripled it, and so on. I am not going to bicker about this - any reasonable person familiar with the lives of mice will understand the figure even if the current format is retained.*

**Response:** We appreciate the Reviewer’s comment which have helped us in improving the manuscript significantly. In accordance with the suggestion by the Reviewer the scale of the Y-axis in the concerned figures has been changed. The Y-axis now represents the body mass of the animal relative to the body mass of the same animal recorded on day 0 (normalised to 100).

The body weight changes has been previously shown as a percentage change out of 100% conventionally for COVID19 animal studies as well as other disease biology studies using animal models. Some of the references of such articles are given below. We used the same formula for calculating the weight changes to maintain the uniformity.

1. Winkler, E.S., Bailey, A.L., Kafai, N.M. *et al.* SARS-CoV-2 infection of human ACE2-transgenic mice causes severe lung inflammation and impaired function. *Nat Immunol* **21**, 1327–1335 (2020). <https://doi.org/10.1038/s41590-020-0778-2>

2. Francis ME, Goncin U, Kroeker A, Swan C, Ralph R, et al. (2021) SARS-CoV-2 infection in the Syrian hamster model causes inflammation as well as type I interferon dysregulation in both respiratory and non-respiratory tissues including the heart and kidney. PLOS Pathogens 17(7): e1009705. <https://doi.org/10.1371/journal.ppat.1009705>

3. Chan JF, Zhang AJ, Yuan S, Poon VK, Chan CC, Lee AC, Chan WM, Fan Z, Tsoi HW, Wen L, Liang R, Cao J, Chen Y, Tang K, Luo C, Cai JP, Kok KH, Chu H, Chan KH, Sridhar S, Chen Z, Chen H, To KK, Yuen KY. Simulation of the Clinical and Pathological Manifestations of Coronavirus Disease 2019 (COVID-19) in a Golden Syrian Hamster Model: Implications for Disease Pathogenesis and Transmissibility. Clin Infect Dis. 2020 Dec 3;71(9):2428-2446. doi: 10.1093/cid/ciaa325. PMID: 32215622; PMCID: PMC7184405.

4. Imai M, Iwatsuki-Horimoto K, Hatta M, Loeber S, Halfmann PJ, Nakajima N, Watanabe T, Ujie M, Takahashi K, Ito M, Yamada S, Fan S, Chiba S, Kuroda M, Guan L, Takada K, Armbrust T, Balogh A, Furusawa Y, Okuda M, Ueki H, Yasuhara A, Sakai-Tagawa Y, Lopes TJS, Kiso M, Yamayoshi S, Kinoshita N, Ohmagari N, Hattori SI, Takeda M, Mitsuya H, Krammer F, Suzuki T, Kawaoka Y. Syrian hamsters as a small animal model for SARS-CoV-2 infection and countermeasure development. Proc Natl Acad Sci U S A. 2020 Jul 14;117(28):16587-16595. doi: 10.1073/pnas.2009799117. Epub 2020 Jun 22. PMID: 32571934; PMCID: PMC7368255.

5. Oh, T.J., Sul, W.J., Oh, H.N. *et al.* Butyrate attenuated fat gain through gut microbiota modulation in db/db mice following dapagliflozin treatment. *Sci Rep* **9**, 20300 (2019). <https://doi.org/10.1038/s41598-019-56684-5>
4. **Comment:** *The language in the original manuscript was generally, in my opinion, clear and of good quality, while the passages that have been added in response to reviewers' comments are rather more variable in quality and would benefit from systematic reading for grammar.*

**Response:** The authors are indebted to the reviewer for the inspiring feedback. The authors have made every effort to make appropriate changes to the language/ grammar, wherever needed.

**Part II –** ***Major Issues:*** *Key Experiments Required for Acceptance*

5. **Comment:** I don't envisage that additional experiments would change the conclusions.

**Part III** – ***Minor Issues:*** *Editorial and Data Presentation Modifications*

6. **Comment:** Already addressed above.

**Reviewer #3**

**Part I –** ***Summary:*** *Strengths/weaknesses of study, novelty/significance, general execution and scholarship*.

1. **Comment:** *The authors report the identification of a broadly neutralizing antibody derived via immunization of mice with the SARS-CoV-2 Alpha RBD. Structural analysis demonstrates this antibody binds to the upper, outer face of the RBD, in a manner that is not impacted by mutations found in VOCs. Therapeutic and protective efficacy for this antibody, P4A2, are described for a variety of SARS-2 CoV variants. Overall, the paper provides sound biochemical, structural and in vivo analyses of this antibody. The authors have, for the most part, addressed all concerns presented during the initial review. There are several additional points that should be addressed to increase the clarity of both text and figures.*

**Response:** The authors are overwhelmed and appreciate the comprehensive critical positive feedback for the betterment of the manuscript. We have revised the manuscript as per all the queries raised.

**Part II –** **Major Issues:** *Key Experiments Required for Acceptance*

**Comment:** No major issues are noted.

**Part III** – **Minor Issues:** *Editorial and Data Presentation Modifications*

2. **Comment:** *The manuscript would generally benefit from editing to improve language use, construction and clarity. There are a number of typos, misplaced or mis-included words throughout the main text and figure legends. For example:*
a. *Line 144: “nearly highly reduced” is awkward and I am not clear what the authors intend to say – “nearly X-fold reduced” or perhaps “highly reduced”?*

**Response:**  The authors are indebted to the reviewers for their sharp observations and are apologetic for the typos and misplaced or mis-included words in the manuscript text. We have corrected these throughout the manuscript text as suggested.

**Previous statement:** Furthermore, as compared to the virus challenge group, mice given P4A2 had *nearly highly reduced* viral RNA levels in their lungs

**Revised statement in the current version of manuscript (Line No: 163-164):** Furthermore, as compared to the virus challenge group, mice given P4A2 had *significantly reduced* viral RNA levels in their lungs

b. *Line 152: should predicated be predicted? If the authors intended to use predicated, I find it difficult to understand.*

**Response:** Line 144 and 152 have been amended as suggested.

c. *Line 156-157: “This might be one probable reason that both antibodies though having the similar affinity to RBD but shows slightly different functional readouts in terms of neutralization (Fig. S8 and S9)” is very difficult to follow. The authors state that Sotrovimab can neutralize the Omicron variant, but with “significantly lesser potency than P4A2.” But then later say “show slightly different functional readouts”. What do the authors mean by “functional readouts” and how does this relate the significantly lesser potency yet also only slightly different function?*

**Response:** We appreciate reviewer’s sharp observations; we have modified the language and we feel that it is now more clear.

**Previous statement:** This might be one probable reason that both antibodies though having a similar affinity to RBD but shows slightly different functional readouts in terms of neutralization

**Revised statement in the current version of manuscript (Line No: 175-176):** Sotrovimab can neutralize the Omicron BA.1 variant, but with significantly lesser potency.

3. ***Comment:*** *Line 161: “the unique mode of RBD recognition employed by P4A2 ensures that this mAb is be able to neutralize new variants that may arise in the future” is too strong of a statement. The virus will continue to evolve and there is no assurance that residues contained within the PA42 epitope will not be mutated in future variants. Indeed, Omicron variant BA.4 is now dominant in many parts of the world and has an F486V mutation, which is a central amino acid in the P4A2 epitope. The authors should discuss (or better yet test) the impact this has on the neutralization profile of P4A2.*

**Response:** Based on the reviewer’s advice, we have toned down the sentence “the unique mode of RBD recognition employed by P4A2 ensures that this mAb is be able to neutralize new variants that may arise in the future” to the following: “Since the P4A2 epitope on the Spike-RBD overlaps with the ACE2 binding site, it is possible that this mAb may be able to neutralize new variants of SARS-CoV-2”.

Regarding the F486V mutation, the aromatic phenylalanine side chain is replaced by a smaller hydrophobic acid valine. Also, the R493Q mutation observed in BA.4/5 may lead to the formation of new interactions with the residues of the light chain of P4A2 Fab. Due to these reasons, P4A2 is expected to exhibit substantial affinity for the Spike-RBD from the Omicron BA.4/5 lineages also. More studies, which are beyond the scope of the present manuscript, are required to ascertain the true impact of the F486V & R493Q mutations on the neutralizing capacity of P4A2.

4. ***Comment:*** *The authors refer to the use of the “Omicron” VOC throughout the text and note in the methods that it is the B.1.1.529 lineage. However, given the rapid expansion of Omicron-based lineages, it would be useful to clarify the mutations in the virus used and indicate the most closely related sub-lineage that the virus used in the studies corresponds to (i.e. BA.1 or otherwise).*

**Response:** We appreciate the Reviewer’s remarks, the correction has been made in the revised manuscript, and mentioned that BA.1 Omicron variant was used for experimental assays throughout the manuscript.

5. **Comment:** *I am also not clear as to how the mode of recognition is unique. Several groups have classified RBD-binding antibodies. Does P4A2 fall into one of these defined groups or is the mode truly different? Likewise, many antibodies that bind in a similar location as P4A2 can bind whether the RBD is up or down. Expansion on the uniqueness or removal of this qualifier would help clarify.*

***Response:*** We agree with the Reviewer’s suggestions and we have removed the unique statement and has modified the same.

**Previous statement:** Overall, our data shows that, the P4A2 mAb has immense therapeutic potential to neutralize the current circulating VOCs. and will be highly effective against future variants as well due to its unique mode of binding.

**Revised statement in the current version of manuscript (Line No: 35-36):** Due to the overlap between the P4A2 epitope and ACE2 binding site on Spike-RBD, P4A2 may also be highly effective against a number of future variants.

**Previous statement:** Therefore, the unique mode of RBD recognition employed by P4A2 ensures that this mAb is be able to neutralize new variants that may arise in the future.

**Revised statement in the current version of manuscript (Line No: 179-180):** Therefore, it is possible that this mAb may be able to neutralize new variants that may arise in the future.

5. **Comment:** *Figure 1A and B: the authors should include the standard deviation values in these tables that reflect the thrice-repeated measurements.*

**Response:** Standard Error of Mean (SEM) values for the triplicate measurement have been incorporated into the tables and also throughout the manuscript (Figure 1A and 1B).

Line No: 252-, 258-259, 320, 329

6. **Comment:** *Figure 1I: the Y-axis label is cut off.*

**Response:** We have corrected the same in the revised manuscript.

7. **Comment:** *Figure 1I and J – what do the error bars indicate? Are these representative graphs with error bars for the technical triplicate measurements or a compilation of all three independent experiments?*

**Response:** The error bars indicate a compilation of all three independent experiments.

8. **Comment:** *Figure 2E: the color scheme, combined with the line and shape choices make it difficult to understand which data points correspond to which conditions. This will be particularly difficult for readers with red-green color blindness. The authors should consider using different shapes and line styles and make these more apparent in the legend.*

**Response:** Figure 2E of the present manuscript has been amended with the suggested changes.

9. **Comment:** *Figure 2F: the legend states that the data is presented as the Log10 N copy number, but the axis is labeled as “Viral load (AU)” and shows powers of 10. The authors may mean to say the axis is shown on a log scale. If not, the values should be simply 1, 6, 8, etc not 101, 106, 108 and the axis label should be “Log Viral load (AU)”. I am also not familiar with what “AU” indicates and how this relates to copy number. There are also some typos in the legend.*

**Response:** Figure 2F of the present manuscript has been modified as per the Reviewer’s suggestion. The typos in the legend text have been corrected.

10. **Comment:** *Figure* *S1B: Include std deviation values.*

**Response:** Standard Error of Mean values have been incorporated into Figure S1B.

11. **Comment:** *Figure S1C: Legend states:*
a. *“RBD-Fc was captured using anti-human Fc biosensor and saturated with P4A2 indicated mAbs at a concentration of 40 μg/ml and unbound P4A2 was washed, followed by incubation with 20 μg/ml of ACE2, P4A2 and P5B3 (binds to a topologically distinct, non-competing epitope and served as a control).”The experimental set up looks like only P4A2 was included as the first antibody (not P4A2 indicated mAbs). P5B3 was included as a control (presumably identified in this same discovery campaign?) but what is P1A2?*

**Response:** We appreciate the Reviewer’s keen observation. We isolated a panel of mAbs in the same study. For better clarity and understanding of readers, we have clarified this in the figure legend and removed the P1A2 data from the figure.

12. **Comment:** *Figure S2: There are many structural models of the RBDs and Spikes from VOCs. Did the authors compare the models they obtained from their simulation studies to those models? Several groups have reported small, but sometimes significant variations in amino acid mainchain and sidechain orientations amongst VOCs, particularly as it relates to antibody binding. Modeling may not accurately reflect the position of the residues modeled here.*

**Response:** The differences in structure of Spike-RBD from different VoCs were taken into account during the modelling exercise. The conformation of the Receptor binding motif from Spike-RBD will be determined by the interaction with the P4A2 Fab and the structure is close to that observed in the complex of Spike-RBD in complex with ACE2. In the absence of P4A2, there will be differences in the conformation adopted by the receptor binding motif, not only between different VoCs, but also between each monomer in the trimeric Spike protein of an individual VoC.

The modelling protocol allows the side chains and backbone atoms of both the epitope and paratope to move for optimal interaction between the antigen epitope and the P4A2 antibody combining site. Since the majority of the differences between the VoCs are in residues that are not part of the epitope, there are only minor differences in the mainchain and sidechain orientations of the interacting residues.

13. **Comment:** *Figure S5: what do the circles indicate?*

Response: We have removed the circles from the figure.

14. **Comment:** *Figure S6: The authors should indicate the model and parameters used for assessment of statistical significance in this figure, as well as any other figure with significance reported*.

**Response:** We have incorporated the details of the statistical models used to analyse the data, throughout the manuscript.

15. **Comment:** *Figure S10: While it is true that none of the residues modeled appear to perturb the binding interface, the statement “None of the mutations perturb the interaction between the P4A2 Fab and the Spike-RBD and therefore will not adversely affect the ability of the mAb to neutralize the SARS-CoV-2 virus” is too presumptive without empirical assessment through structural or in vitro analysis. The authors should also include residues identified in newly emergent Omicron lineages, such as BA.2 and BA.4, particularly the F486V mutation.*

**Response:** Based on the reviewer’s advice, we have toned down the sentence ““None of the mutations perturb the interaction between the P4A2 Fab and the Spike-RBD and therefore will not adversely affect the ability of the mAb to neutralize the SARS-CoV-2 virus” and changed it to “None of the predicted mutations of the Spike-RBD overlap with the residues that interact with P4A2 Fab and therefore it is possible that these mutations may not reduce the ability of P4A2 to neutralize the corresponding new variants of SARS-CoV-2”

A new figure regarding the mutations observed in the BA.1, BA.2, BA.3, BA.4/5, BA.2.75, BA.2.1.21 Omicron lineages is added as Figure S11. We have also added the following sentences in the manuscript “Recently new lineages of Omicron have emerged namely BA.2, BA.2.75, BA.2.12.1BA.3, BA.4, and BA.5 [6] (Fig S10). The 486Phe residue in BA.4 and BA.5 is substituted with Val. Since the side chain of Val is also hydrophobic P4A2 may retain substantial affinity for Spike-RBD. Also as expected, 486PheVal results in reduction of affinity of Spike-RBD for ACE2 receptor, but the R493Q reversion mutation reinstates the affinity [7]. R493Q may also attenuate the possible reduction in affinity of Spike-RBD for P4A2 due to F486V since the shorter Gln side chain will be closer to the paratope surface and can form new hydrogen bonds with residues of CDR L1 of P4A2. P4A2 may therefore, retain significant capacity to bind to Spike protein from BA.4/5 and further studies are required to ascertain the ability of P4A2 to neutralize these strains.”

**17.Comment:** *Figure S11:*

a. *The legend states: “The mAbs JMB2002 and S3H3 are now shown here ...” Do the authors mean to say these mAbs are NOT shown here (as they are not).*

**Response:** We appreciate reviewers keen observation and we have corrected the sentence.

b. *The omission of Sotrovimab is interesting since the authors specifically show data for this antibody. Is there a reason it was not included?*

**Response:** The authors accept we missed adding the same and we have included the same in the revised manuscript.

c. *The figure would benefit from a reorganization that groups the RBDs by their orientation.*

**Response:** Authors appreciate the Reviewer’s recommendation, Figure S11 has been accordingly reorganized and incorporated into the revised manuscript.

18. **Comment:** *ELISA section of Methods: ug/mL-1 should be changed to ug/mL or ug mL-1..*

**Response:** The unit has been corrected in the revised manuscript.
